# Renal IGFBP6 Interacts With THBS1 to Drive Renal Cellular Senescence and Fibrosis

**DOI:** 10.1002/advs.75802

**Published:** 2026-05-22

**Authors:** Ju‐tao Yu, Chang‐geng Xu, Xiao‐wei Hu, Run‐run Shan, Jia‐nan Wang, Xiang‐yu Li, Yu‐qing Wang, Yu‐hang Dong, Shen‐Zhuo‐Fan Huang, Shuai Sun, Tian Zhao, Ya‐Xin Ru, Xiao‐yu Shen, Rui Hou, Jian‐min You, Fei Zhang, Chao Li, Jie Wei, Chao Hou, Zhi‐juan Wang, Dan‐feng Zhang, Juan Jin, Jia‐gen Wen, Yu‐jie Liu, Ming‐ming Liu, Xiao‐ming Meng, Wei Wang

**Affiliations:** ^1^ Inflammation and Immune Mediated Diseases Laboratory of Anhui Province the Key Laboratory of Anti‐inflammatory of Immune Medicines Ministry of Education Anhui Institute of Innovative Drugs School of Pharmacy Anhui Medical University Hefei China; ^2^ Department of Urology The Central Hospital of Wuhan Tongji Medical College Huazhong University of Science and Technology Wuhan Hubei China; ^3^ Key Laboratory for Molecular Diagnosis of Hubei Province The Central Hospital of Wuhan Tongji Medical College Huazhong University of Science and Technology Wuhan Hubei China; ^4^ Department of Clinical Pharmacy Anhui Provincial Children's Hospital Hefei China; ^5^ State Key Laboratory of Pharmaceutical Biotechnology College of Life Sciences Nanjing University Nanjing China; ^6^ Department of Urology The First Affiliated Hospital of Anhui Medical University Institute of Urology, and Anhui Province Key Laboratory of Urological and Andrological Diseases Research and Medical Transformation Anhui Medical University Hefei China; ^7^ School of Chinese Medicine Li Ka Shing Faculty of Medicine The University of Hong Kong Hong Kong China; ^8^ Department of Nephrology The Second Affiliated Hospital of Anhui Medical University Hefei Anhui China; ^9^ Department of Pharmacology School of Basic Medical Sciences Key Laboratory of Anti‐Inflammatory and Immunopharmacology Ministry of Education Anhui Medical University Hefei China; ^10^ Department of Obstetrics and Gynecology The First Affiliated Hospital of USTC University of Science and Technology of China Hefei Anhui China; ^11^ Department of Urology The Fifth Affiliated Hospital of Anhui Medical University Fuyang China

**Keywords:** cellular senescence, CD47, IGFBP6, renal fibrosis, THBS1

## Abstract

Epithelial dedifferentiation and myofibroblast activation are critical drivers of chronic kidney disease (CKD) progression. Elevated levels of IGFBP6 have been linked to decreased renal function in CKD patients, but its precise role and underlying mechanisms remain unclear. In this study, we observed significantly increased IGFBP6 expression in the kidney tissues of both renal fibrosis patients and animal models. Global or tubule‐specific IGFBP6 knockout attenuated renal cellular senescence and fibrosis development in mice. In vitro, IGFBP6 deficiency preserved epithelial cell phenotype and inhibited fibroblast activation. Additionally, anti‐IGFBP6 treatment demonstrated promising therapeutic effects in alleviating renal cellular senescence and fibrosis. Mechanistically, IGFBP6, acting as an adaptor protein, could bind to thrombospondin 1 (THBS1) and prevent its ubiquitination‐mediated degradation, thereby activating the THBS1‐CD47 cellular pathway in epithelial cells, which contributed to renal cellular senescence and fibrosis. Notably, both genetic and neutralizing antibody‐mediated inhibition of IGFBP6 alleviated renal cellular senescence and fibrosis, suggesting that the IGFBP6/THBS1/CD47 axis represents a potential therapeutic target for chronic kidney injury.

## Abstract 2

IGFBP6 is upregulated in human and murine fibrotic kidneys. IGFBP6 deletion or inhibition reduces tubular epithelial dedifferentiation, cellular senescence, and renal fibrosis. Mechanistically, IGFBP6 stabilizes THBS1 to activate the CD47 pathway, establishing the IGFBP6/THBS1/CD47 axis as a novel therapeutic target for chronic kidney disease.

## 1 Introduction

Chronic kidney disease (CKD) is a worldwide health issue with increasing rates, impacting about 10% of people globally [1]. Renal fibrosis is a common pathological feature in CKD, and its severity is closely correlated with patient prognosis. The main features of renal fibrosis include the infiltration of inflammatory cells, activation of myofibroblasts, and accumulation of interstitial collagen [2]. Proximal tubular cells (PTCs) have long been recognized as primary initiators of renal fibrosis due to their high sensitivity to injury [3]. Initially, injured PTCs exhibit maladaptive responses, secreting various cytokines and growth factors that amplify the inflammatory response. Subsequently, this inflammatory cascade stimulates fibroblast activation and induces PTCs to undergo partial epithelial‐mesenchymal transition (EMT), leading to the production of excessive extracellular matrix (ECM) components. This ultimately results in renal fibrosis and a consequent decline in renal function [4, 5]. In recent years, increasing evidence suggests that the senescence of renal tubular epithelial cells plays a significant role in the pathogenesis of kidney fibrosis [6]. Senescent renal tubular epithelial cells exhibit a senescence‐associated secretory phenotype (SASP), characterized by the production of profibrotic cytokines that promote fibrosis development [7]. Emerging studies have indicated that enhanced senescence of PTCs is a hallmark of renal fibrosis and contributes to its progression [7]. However, the precise mechanisms underlying renal aging remain poorly understood. Therefore, identifying novel therapeutic targets and elucidating specific mechanisms that prevent proximal tubular cell senescence and subsequent myofibroblast activation may offer promising therapeutic strategies for renal fibrosis.

Insulin‐like growth factor binding protein 6 (IGFBP6) is a member of the IGFBP family, a group of secreted proteins capable of interacting with IGFs/IGFR. Notably, IGFBP6 can also exert its functions in various biological processes independently of IGFs/IGFR signaling, including cellular growth, differentiation [8] and immune regulation [9]. Previous studies have reported significantly elevated serum levels of IGFBP6 in patients with CKD [10, 11, 12] or end‐stage renal disease (ERSD) [12]. Additionally, IGFBP6 expression has been shown to correlate negatively with estimated glomerular filtration rate (eGFR). The expression of IGFBP6 also significantly increased in fluorescence‐activated cell sorting (FACS)‐isolated α‐smooth muscle actin (αSMA)^+^ cells from fibrotic kidneys of αSMA‐RFP transgenic mice [13]. Collectively, these findings suggest a crucial role for IGFBP6 in renal fibrosis. However, the exact function and regulatory mechanisms in kidney fibrosis are yet to be clarified.

In this study, we identified sustained high levels of IGFBP6 mRNA and protein expression in the fibrotic kidneys of both human and mouse models. These levels were positively correlated with CKD severity and renal fibrosis. Moreover, using IGFBP6 knockout (KO) mice and IGFBP6 tubular epithelial cell conditional knockout (cKO) mice, we confirmed that IGFBP6 could promote fibrogenesis and myofibroblast activation in renal cellular senescence and fibrosis. Mechanistically, we found that IGFBP6 could bind to thrombospondin‐1 (THBS1), inhibiting its ubiquitination and degradation. This led to the activation of the THBS1‐CD47 cellular pathway in epithelial cells, resulting in tubular cell senescence. Importantly, we validated the anti‐cellular senescence and fibrotic effects of IGFBP6‐neutralizing antibodies in multiple renal fibrosis models, suggesting that IGFBP6 may be a novel therapeutic target for CKD.

## 2 Results

### 2.1 Elevated H3K4me3‐Dependent IGFBP6 Expression Correlated with the Severity of Renal Fibrosis and Dysfunction

**FIGURE 1:**
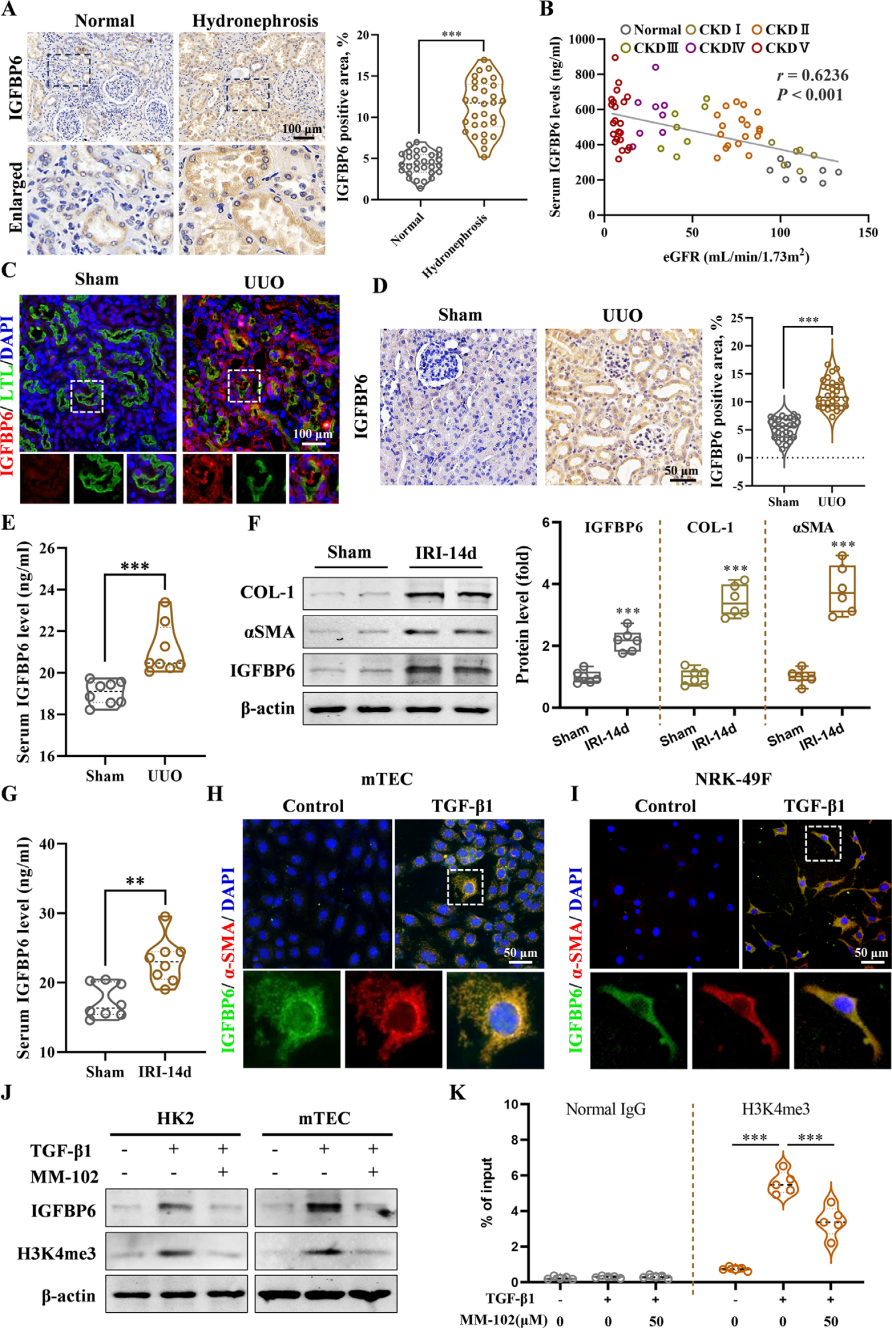
H3K4me3 activation in CKD leads to increased expression of IGFBP6. (A) Representative IHC staining of IGFBP6 in kidney biopsies from CKD patients (n = 30 views of 6 mice/group). (B) Human Insulin‐like growth factor‐binding protein 6 ELISA assay. Serum IGFBP6 level is increased in patients with different stages of CKD and negatively correlated with glomerular filtration rate (n = 69). (C) Representative immunofluorescence (IF) staining of IGFBP6 and LTL in UUO mice model. LTL was employed to stain proximal tubules (n = 6 mice/group). (D) Representative IHC staining of IGFBP6 in UUO mice model (n = 30 views of 6 mice/group). (E) Serum IGFBP6 levels in the UUO mouse model. (F) Protein level of COL‐1, αSMA, and IGFBP6 in IRI‐14d‐induced mice model (n = 6 mice/group). (G) Serum IGFBP6 levels in IRI‐14d‐induced mouse model. (H‐I) Representative immunofluorescence (IF) staining of IGFBP6 and αSMA in TGF‐β1‐induced mTEC and NRK‐49F cells model. (J) Western blot analyses of H3K4me3 and IGFBP6 in HK2 or mTEC cells with MM‐102 stimulation. (K) The results of chromatin immunoprecipitation (ChIP) assay showed that MM‐102 stimulation reduced H3K4me3 binding in the IGFBP6 promoter region in HK2 cells with TGF‐β1 stimulation. Data are presented as mean ± SEM from at least 3–4 independent in vitro experiments and 6–8 mice in vivo. Statistical differences were determined using an independent sample t‐test and one‐way ANOVA with Tukey's post hoc analysis. ** *p* < 0.01, ***p < 0.001.

Immunohistochemistry (IHC) staining and Western blot analysis showed that the notion of elevated IGFBP6 levels in kidney tissues from CKD patients (Figure 1A; Figure S1A). In addition, serum IGFBP6 levels were found to be elevated in CKD patients and negatively correlated with eGFR (Figure 1B). First, we found that IGFBP6 expression was significantly increased in unilateral ureteral obstruction (UUO)‐induced renal fibrosis model. Immunofluorescence (IF) staining with Lotus tetragonolobus lectin (LTL), a proximal tubule marker, confirmed that IGFBP6 was primarily localized in the tubules (Figure 1C,D; Figure S1B‐D). ELISA experiments further demonstrated a significant increase in IGFBP6 content in the UUO mouse model (Figure 1E). Consistent with the UUO model, IRI‐14d‐induced renal fibrosis also exhibited increased IGFBP6 expression and secretion (Figure 1F,G).

Given the established roles of proximal tubular epithelial cells and fibroblasts in renal fibrogenesis [14, 15], we examined IGFBP6 regulation in these cell types under TGF‐β1 stimulation. Concentration‐dependent increases in IGFBP6 expression paralleling α‐smooth muscle actin (αSMA) upregulation was observed through IF and Western blot analyses in both epithelial and fibroblast models (Figure 1H,I; Figure S1E,F).

To investigate the molecular basis of IGFBP6 elevation during fibrosis, we performed promoter analysis using the UCSC Genome Browser (http://genome.ucsc.edu/) [16]. The IGFBP6 promoter region displayed substantial H3K4 trimethylation (H3K4me3) signals (Figure S2A), suggesting epigenetic regulation. As the H3K4me3 methyltransferase MLL1 requires WDR5 for catalytic activity [17], we employed two well‐characterized MLL1/WDR5 complex inhibitors (MM‐102 and OICR‐9429) [18, 19, 20]. Both compounds induced time‐ and dose‐dependent reductions in H3K4me3 levels and corresponding decreases in IGFBP6, MLL1, and WDR5 expression at mRNA and protein levels (Figure 1J; Figure S2B‐E). Chromatin immunoprecipitation (ChIP) assays confirmed H3K4me3 enrichment at the IGFBP6 promoter, with significant signal attenuation following MLL1/WDR5 inhibition (Figure 1K). These findings collectively demonstrate that MLL1/WDR5‐mediated H3K4me3 modification contributes to IGFBP6 transcriptional activation during fibrogenesis (Figure S2F). Our multi‐model analysis establishes renal IGFBP6 expression as a consistent biomarker correlating with tubulointerstitial fibrosis progression.

### 2.2 IGFBP6‐Mediated Progression of Renal Fibrosis in Mice

**FIGURE 2:**
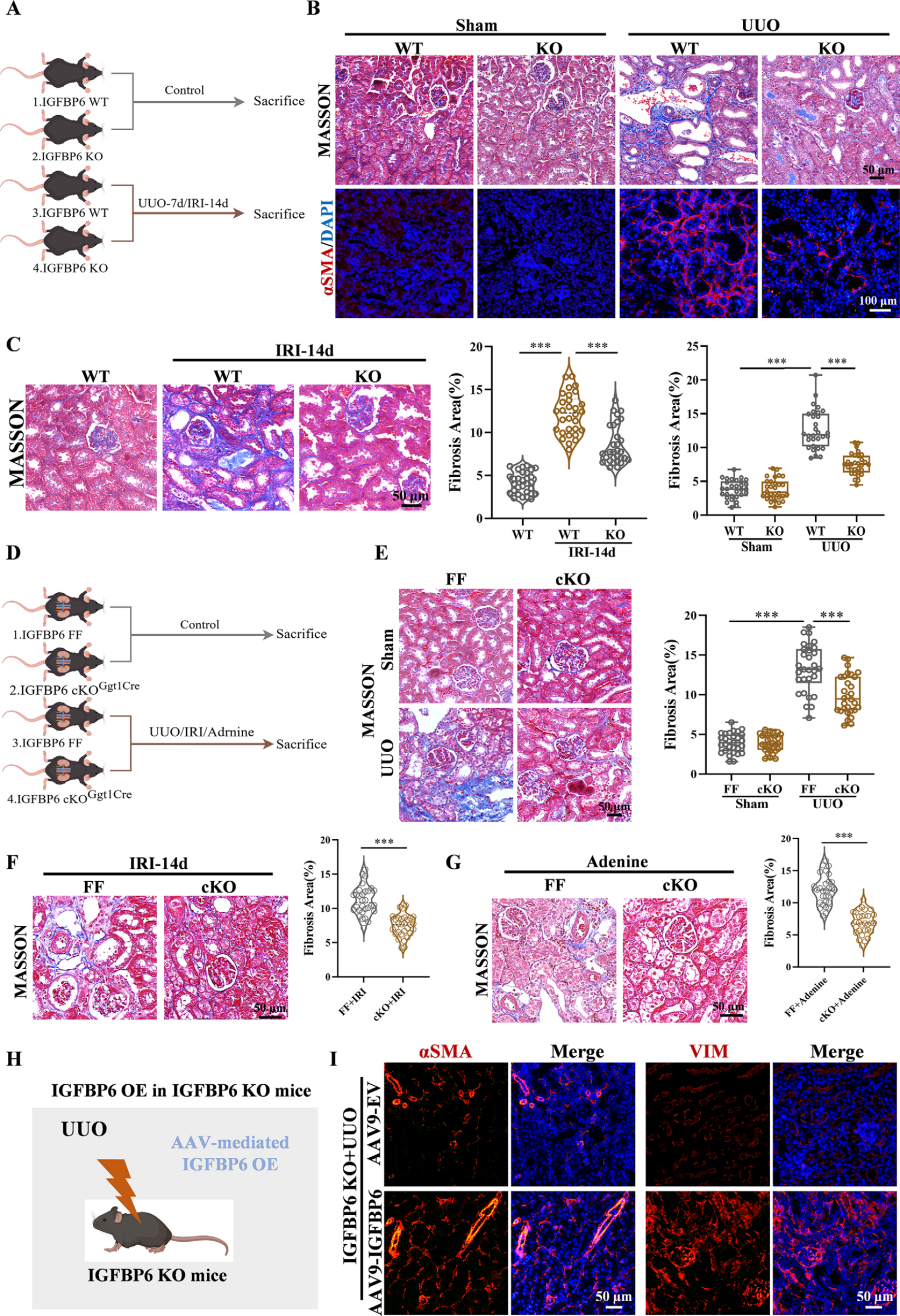
IGFBP6 promotes renal fibrosis. (A) Established UUO and IRI induced renal fibrosis model on IGFBP6 global knockout mice. (B) Representative Masson and immunofluorescence staining of kidneys from WT and IGFBP6‐KO mice treated with UUO. (C) Representative Masson staining pictures of kidneys from WT and IGFBP6‐KO mice (n = 6 mice/group). (D) Established UUO, IRI, and adenine induced renal fibrosis model on IGFBP6 tubule‐conditional knockout mice. (E) Representative Masson staining pictures of kidneys in UUO‐treated IGFBP6‐cKO mice (n = 6 mice/group). (F‐G) Representative Masson staining of IRI and adenine induced renal fibrosis model in cKO mice (n = 6 mice/group). (H) Injected AAV9‐mediated IGFBP6 OE into the tail vein of IGFBP6 globally knockout mice. (I) Immunofluorescence staining and quantification of αSMA and VIM from AAV9‐mediated IGFBP6 overexpression in KO mice (n = 30 views of 6 mice/group). Data are presented as mean ± SEM from at least 6–8 mice in vivo. Statistical differences were determined using an independent sample t‐test and one‐way ANOVA with Tukey's post hoc analysis. *** *p* < 0.001.

To investigate IGFBP6's functional contribution to renal fibrosis, we established global IGFBP6 knockout (KO) mice validated through PCR genotyping and immunofluorescence confirmation of complete IGFBP6 ablation (Figure S3A,B). Comparative analysis of UUO‐ and ischemia‐reperfusion injury (IRI)‐induced fibrosis models in wild‐type (WT) versus KO mice (Figure 2A) demonstrated substantial fibrotic attenuation in KO animals. Masson's trichrome staining and immunofluorescence quantification revealed marked reductions in collagen deposition and fibrotic markers in KO kidneys (Figure 2B; Figure S3D). Western blot and IHC analyses further confirmed significant suppression of UUO‐induced fibrotic progression in KO mice (Figure S3C,E). This protective effect extended to IRI models, with KO animals showing significantly mitigated renal damage (Figure 2C; Figure S3F,G).

To specifically investigate the role of IGFBP6 in renal proximal tubules, we generated a mouse model with conditional knockout of IGFBP6 specifically in these cells (Figure S4A,B). We subsequently constructed three fibrosis models by UUO, IRI‐14d and adenine in IGFBP6^floxflox^ (FF) and renal tubules conditional knockout (cKO) mice (Figure 2D). Masson's trichrome staining revealed a trend toward amelioration of UUO‐induced renal fibrosis in IGFBP6 cKO tissues compared to FF control tissues (Figure 2E). Furthermore, IGFBP6 cKO significantly decreased the protein levels of markers associated with renal fibrosis, including collagen I (COL‐1), αSMA, and vimentin (VIM) (Figure S4C,D). Importantly, IGFBP6 cKO also alleviated renal fibrosis in IRI and adenine‐induced renal fibrosis models (Figure 2F,G).

To further assess the impact of IGFBP6 on renal fibrosis, we restored IGFBP6 expression in IGFBP6 knockout mice using an AAV‐packaged IGFBP6 overexpression (OE) plasmid, followed by the induction of UUO (Figure 2H). Western blot and IF confirmed successful overexpression of IGFBP6 in the IGFBP6 KO mice (Figure S4E,F). Masson's trichrome staining, Western blot, and immunofluorescence staining revealed that the level of fibrosis in UUO mice with restored IGFBP6 expression was exacerbated compared to control mice (Figure 2I; Figure S4G‐I). Collectively, our results indicate that ectopic expression of IGFBP6 can promote the progression of renal fibrosis in vivo.

### 2.3 IGFBP6 Promotes Fibrosis in Tubular Epithelial Cells and Fibroblasts

**FIGURE 3:**
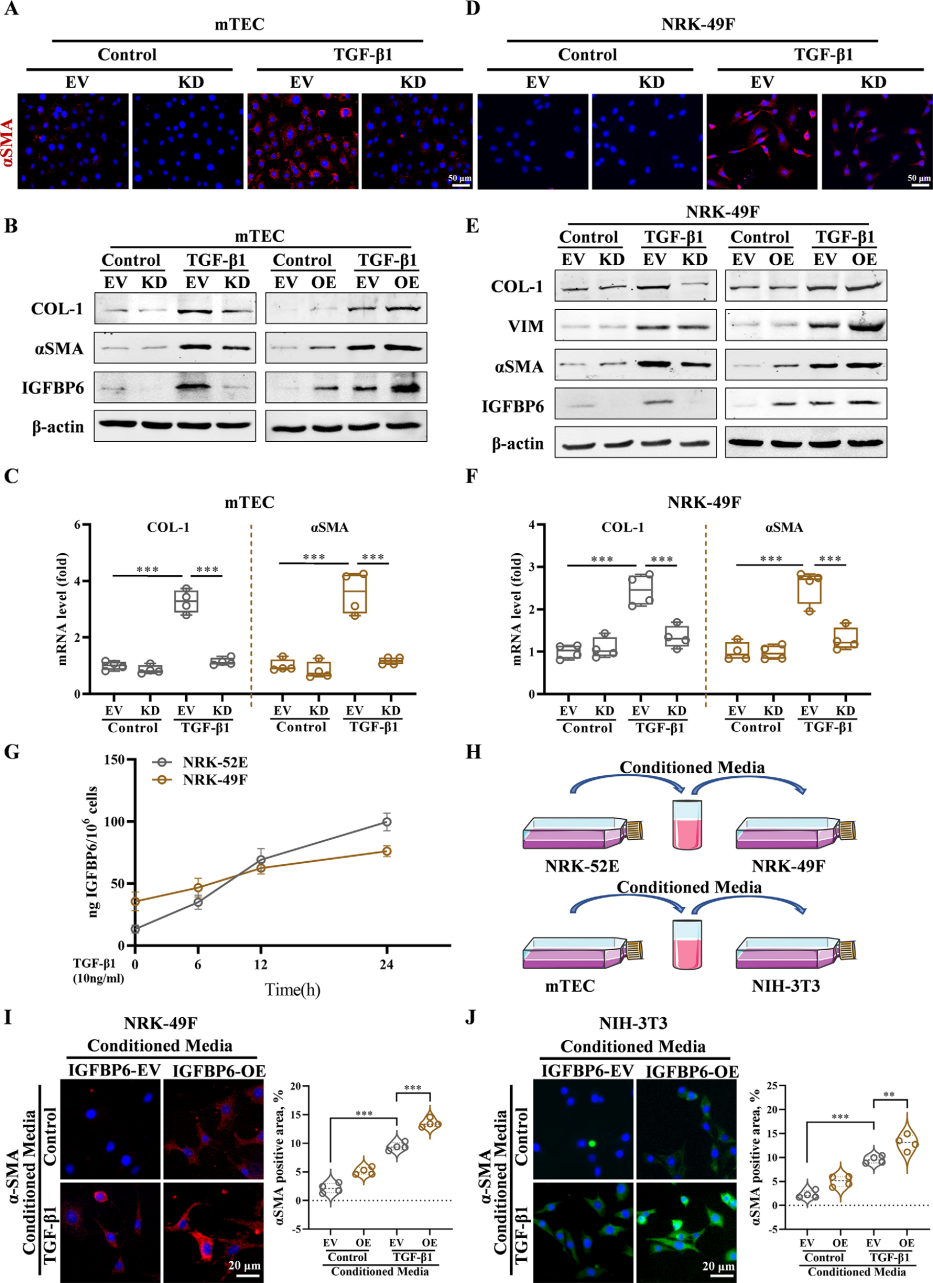
In vitro silencing/overexpression of IGFBP6 reduces/exacerbates the fibrotic response of renal tubular epithelial cells and fibroblasts. (A) Immunofluorescence staining of αSMA in mTEC cells. (B‐C) Protein and mRNA levels of COL‐1, αSMA, and IGFBP6 in mTEC cells. (D) Immunofluorescence staining of αSMA in NRK‐49F cells. (E‐F) Protein and mRNA levels of COL‐1, αSMA, VIM, and IGFBP6 in NRK‐49F cells. (G) Rat IGFBP6 ELISA assay. Under the stimulation of TGF‐β1, the rate and content of IGFBP6 secreted by rat renal TEC NRK‐52E were higher than rat renal fibroblast cell NRK‐49F. (H) Culture of fibroblast cells using a conditioned medium of TEC overexpressing IGFBP6. (I) Immunofluorescence staining of αSMA in NRK‐49F cells. (J) Immunofluorescence staining of αSMA in NIH‐3T3 cells. Data are presented as mean ± SEM from at least 3–4 independent in vitro experiments. Statistical differences were determined using an independent sample t‐test and one‐way ANOVA with Tukey's post hoc analysis. **p < 0.01, ***p < 0.001.

Epithelial injury and the activation of myofibroblasts play a vital role in CKD pathogenesis [21]. To validate our in vivo findings, we first evaluated the function of IGFBP6 in murine tubular epithelial cells (mTECs) and NRK‐49F cells in response to TGF‐β1 stimulation. Immunofluorescence, Western blotting, and real‐time PCR analyses revealed that IGFBP6 silencing attenuated TGF‐β1‐induced fibrosis, while IGFBP6 overexpression exacerbated fibrosis in both epithelial cells and fibroblasts (Figure 3A‐F; Figures S5A‐E and S6A‐E). In addition, we further found that silencing IGFBP6 in primary tubular cells also alleviated TGF‐β1‐induced fibrosis‐like response (Figure S5F). Importantly, as a secreted protein, we examined IGFBP6 levels in the supernatant of both cell types after TGF‐β1 stimulation. Interestingly, ELISA results indicated that fibroblasts secreted higher levels of IGFBP6 than epithelial cells under normal conditions. However, upon TGF‐β1 stimulation, epithelial cells exhibited a significantly higher secretion rate of IGFBP6 compared to fibroblasts (Figure 3G). Based on these findings, we hypothesized that IGFBP6 produced and secreted by epithelial cells could enter fibroblasts and enhance fibrosis signaling. To validate this hypothesis, we stimulated rat and mouse fibroblasts (NRK‐49F and NIH‐3T3) with conditioned media from IGFBP6‐overexpressing rat and mouse renal tubular epithelial cells (NRK‐52E and mTEC), respectively (Figure 3H). Immunofluorescence analysis demonstrated that stable overexpression of IGFBP6 in epithelial cells led to increased fibroblast activation, as evidenced by the upregulation of αSMA expression, in comparison to control media (Figure 3I,J). Additionally, we observed that the addition of recombinant IGFBP6 protein to both epithelial cells and fibroblasts resulted in the upregulation of COL‐1 and αSMA expression, exacerbating fibrosis in a concentration‐dependent manner (Figures S5G and S6F). In conclusion, our results demonstrate that TGF‐β1 stimulation rapidly increases IGFBP6 expression and secretion in epithelial cells, which in turn promotes both autocrine and paracrine fibrosis signaling, ultimately leading to global renal fibrosis.

### 2.4 IGFBP6 Promotes Cell Senescence of Renal Tubular Epithelial Cells

**FIGURE 4:**
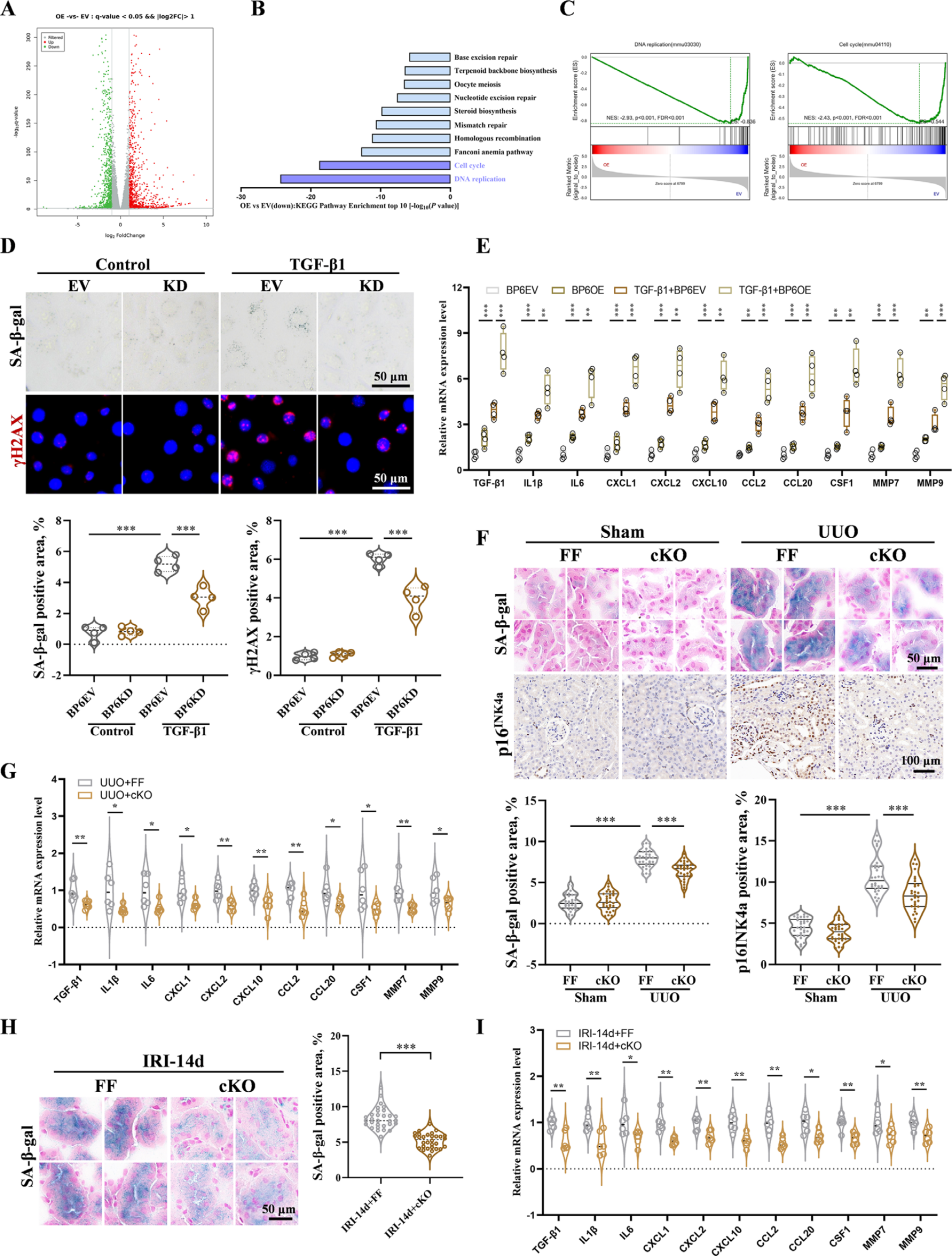
IGFBP6 promotes cell senescence. (A) Volcano plots. Comparing the upregulated and downregulated proteins in mTEC with IGFBP6 overexpress. (B) KEGG pathway enrichment top 10 in mTEC with IGFBP6 overexpress. (C) GSEA analysis of differentially expressed genes in IGFBP6 overexpress mTEC cells shows significant enrichment of the cell cycle and DNA replication pathway. (D) SA‐β‐gal staining and immunofluorescence staining of γH2AX in IGFBP6‐silencing mTEC cells with or without TGF‐β1 stimulation (n = 4). (E) The mRNA level of SASP‐related inflammatory factors in IGFBP6‐overexpressing mTEC cells with TGF‐β1 stimulation (n = 4). (F) The IHC of p16^INK4a^ and SA‐β‐gal staining in UUO‐treated IGFBP6‐cKO mice (n = 30 views of 6 mice/group). (G) The mRNA level of SASP‐related inflammatory factors in UUO‐treated IGFBP6‐cKO mice (n = 6 mice). (H) The SA‐β‐gal staining in IRI‐14d‐treated IGFBP6‐cKO mice (n = 30 views of 6 mice/group). (I) The mRNA level of SASP‐related inflammatory factors in IRI‐14d‐treated IGFBP6‐cKO mice (n = 6 mice). Data are presented as mean ± SEM from at least 3–4 independent in vitro experiments and 6–8 mice in vivo. Statistical differences were determined using an independent sample t‐test and one‐way ANOVA with Tukey's post hoc analysis. *p < 0.05, **p < 0.01, ***p < 0.001.

To further investigate the mechanism of IGFBP6 in renal fibrosis, we performed RNA‐seq analysis. As shown in Figure 4A, a total of 2059 differentially expressed genes were identified between control and IGFBP6‐overexpressing mTEC cells, with 1126 genes upregulated and 933 genes downregulated. Cell senescence is closely linked to renal fibrosis, characterized by cell cycle arrest, impaired DNA repair, and increased inflammatory factors [22, 23, 24]. Interestingly, KEGG analysis and GSEA revealed that IGFBP6 was negatively correlated with cell cycle and DNA repair pathways (Figure 4B,C; Figure S7A,B). Consistently, IGFBP6 significantly upregulated inflammation‐related signaling pathways (Figure S7C,D). Taken together, these data suggest a potential link between IGFBP6 and the regulation of cell cycle, DNA replication, and SASP‐related inflammatory factors, implicating a role for IGFBP6 in cellular senescence. To explore this further, we examined the impact of IGFBP6 on cellular senescence using SA‐β‐gal staining, a classic marker of senescence [7]. Additionally, we assessed the expression of γH2AX, a phosphorylated form of histone H2AX involved in DNA damage repair and cell cycle regulation [25]. Our results demonstrated that silencing IGFBP6 significantly reduced the elevation of cellular senescence levels in TGF‐β1‐stimulated mTEC cells (Figure 4D). Similar results were obtained in primary renal tubular epithelial cells (Figure S7E). Correspondingly, real‐time PCR analysis revealed that IGFBP6 overexpression increased the expression of SASP‐related inflammatory factors in TGF‐β1‐induced HK2 cells (Figure 4E). The presence of p16INK4a‐positive cells in the kidney is considered a molecular marker of renal senescence [26]. Consistently, we found that IGFBP6 cKO significantly mitigated UUO‐induced cell senescence, a finding that was also observed in the IRI‐14d‐induced renal fibrosis model (Figure 4F‐I). Overall, our findings suggest that IGFBP6 may act as a critical promoter of cellular senescence, contributing to the development of renal fibrosis.

### 2.5 IGFBP6 Binds to THBS1 and Antagonizes Its Ubiquitination Degradation by E3 Ubiquitin Ligase NEDD4

**FIGURE 5:**
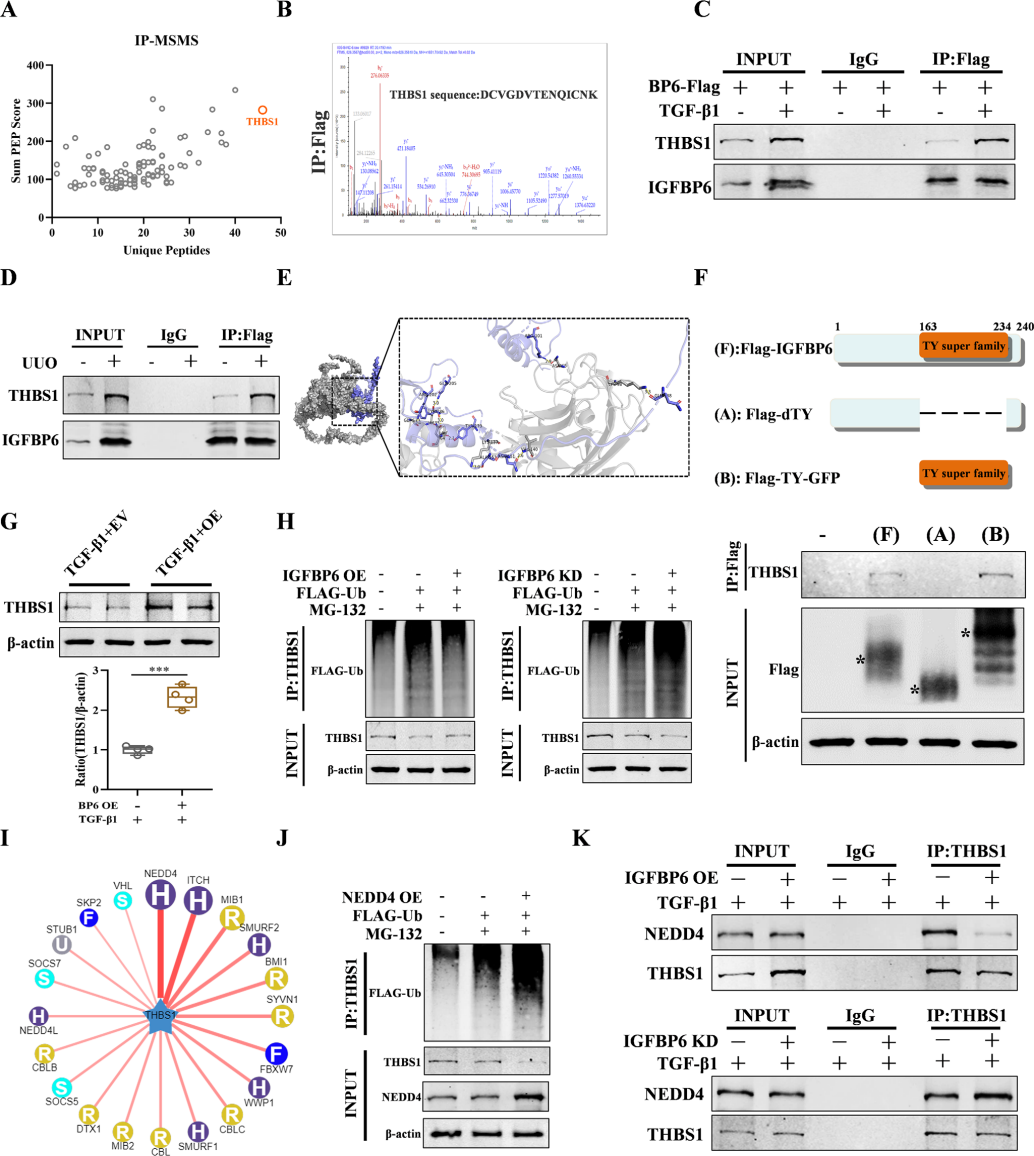
IGFBP6 binds to THBS1 and antagonizes its ubiquitination degradation by E3 ubiquitin ligase NEDD4. (A‐B) Immunoprecipitation coupled with mass spectrometry (IP‐MS/MS) assay. Results showed that the binding score and unique peptides of insulin‐like growth factor‐binding protein 6 (IGFBP6) and THBS1 were relatively high in TGF‐β1‐induced IGFBP6‐OE HK2 cells. (C) Co‐immunoprecipitation (Co‐IP) assay detects the interactions of IGFBP6 and THBS1 in TGF‐β1‐treated HK2 cells. (D) Co‐immunoprecipitation (Co‐IP) assay detects the interactions of IGFBP6 and THBS1 in UUO mice model. (E) Molecular docking of IGFBP6 with THBS1. (F) Full‐length flag‐IGFBP6, TY deletion mutant (dTY) or TY construct (TY) were expressed in 293T cells, and Co‐IP was performed with anti‐Flag antibody. (G) The western blot analyses of THBS1 in TGF‐β1‐treated IGFBP6‐OE HK2 cells. (H) The co‐expression of IGFBP6 OE or KD and FLAG‐Ub in HK2 cells was performed with or without MG‐132. THBS1 was isolated by IP, and its ubiquitination levels were measured using anti‐FLAG antibodies. (I) The UbiBrowser assay results indicate that NEDD4 is the key E3 ubiquitin ligase for THBS1. (J) Co‐IP assay detects the effect of NEDD4 overexpressing on THBS1 ubiquitination level in HK2 cells. (K) IGFBP6 OE or siRNA and FLAG‐Ub were co‐expressed and applied with MG‐132 in HK2 cells. THBS1 was isolated by IP, and NEDD4 levels were assessed with anti‐NEDD4 antibody. Data are presented as mean ± SEM from at least 3–4 independent in vitro experiments and 6–8 mice in vivo. Statistical differences were determined using an independent sample t‐test and one‐way ANOVA with Tukey's post hoc analysis. **p < 0.01.

Given the pivotal role of IGFBP6 in driving renal senescence and fibrosis, we sought to investigate the underlying mechanisms. To identify potential protein interactors of IGFBP6 and explore their regulatory mechanisms in HK2 cells, we employed mass spectrometry analysis. This analysis revealed THBS1 as a strong candidate interacting partner of IGFBP6 in TGF‐β1‐induced HK2 cells, with high scores and unique peptides (Figure 5A,B). THBS1 has been implicated in regulating cell senescence and fibrosis progression [27, 28, 29]. Co‐immunoprecipitation (Co‐IP) experiments demonstrated a significant enhancement of the IGFBP6‐THBS1 interaction in TGF‐β1‐stimulated HK2 cells (Figure 5C). Consistent with these in vitro findings, increased binding of IGFBP6 to THBS1 was observed in vivo in the UUO model (Figure 5D). To further delineate the specific functional domains involved in the IGFBP6‐THBS1 interaction, we employed molecular docking analysis, which predicted that the TY superfamily domain of IGFBP6 is the primary binding site for THBS1 (Figure 5E; Figure S8A). To validate this hypothesis, we generated truncated mutants of IGFBP6 and THBS1 for Co‐IP assays. THBS1 interacted with full‐length Flag‐IGFBP6, Flag‐dTY, and Flag‐TY‐GFP in TGF‐β1‐treated HK2 cells, indicating that the TY domain of IGFBP6 is indeed the primary binding site for THBS1 (Figure 5F).

Interestingly, IGFBP6 overexpression resulted in increased THBS1 protein expression in our experiments (Figure 5G). This finding led us to hypothesize that IGFBP6 might antagonize the ubiquitination‐mediated degradation pathway of THBS1, thereby increasing its protein levels. To validate this hypothesis, we observed a decrease in THBS1 ubiquitination upon IGFBP6 overexpression and an increase upon silencing (Figure 5H). To further investigate the potential role of IGFBP6 in THBS1 ubiquitination, we utilized UbiBrowser (http://ubibrowser.ncpsb.org) to predict the ubiquitination region of THBS1. The analysis indicated that the E3 ubiquitin ligase NEDD4 had the highest score and exhibited a significant overlap with the binding regions of both IGFBP6 and THBS1 (Figure 5I; Figure S8B). Subsequently, we overexpressed NEDD4 and observed a significant increase in THBS1 ubiquitination levels (Figure 5J). In addition, Co‐IP experiments revealed that IGFBP6 overexpression significantly reduced the interaction between NEDD4 and THBS1, whereas IGFBP6 silencing markedly enhanced their binding (Figure 5K). These findings collectively support the hypothesis that IGFBP6 binds to THBS1 through the TY superfamily functional domain and antagonizes its ubiquitination degradation mediated by the E3 ubiquitin ligase NEDD4.

### 2.6 IGFBP6 Promotes Cellular Senescence and Renal Fibrosis by Promoting THBS1 Binding to the CD47 Receptor

**FIGURE 6:**
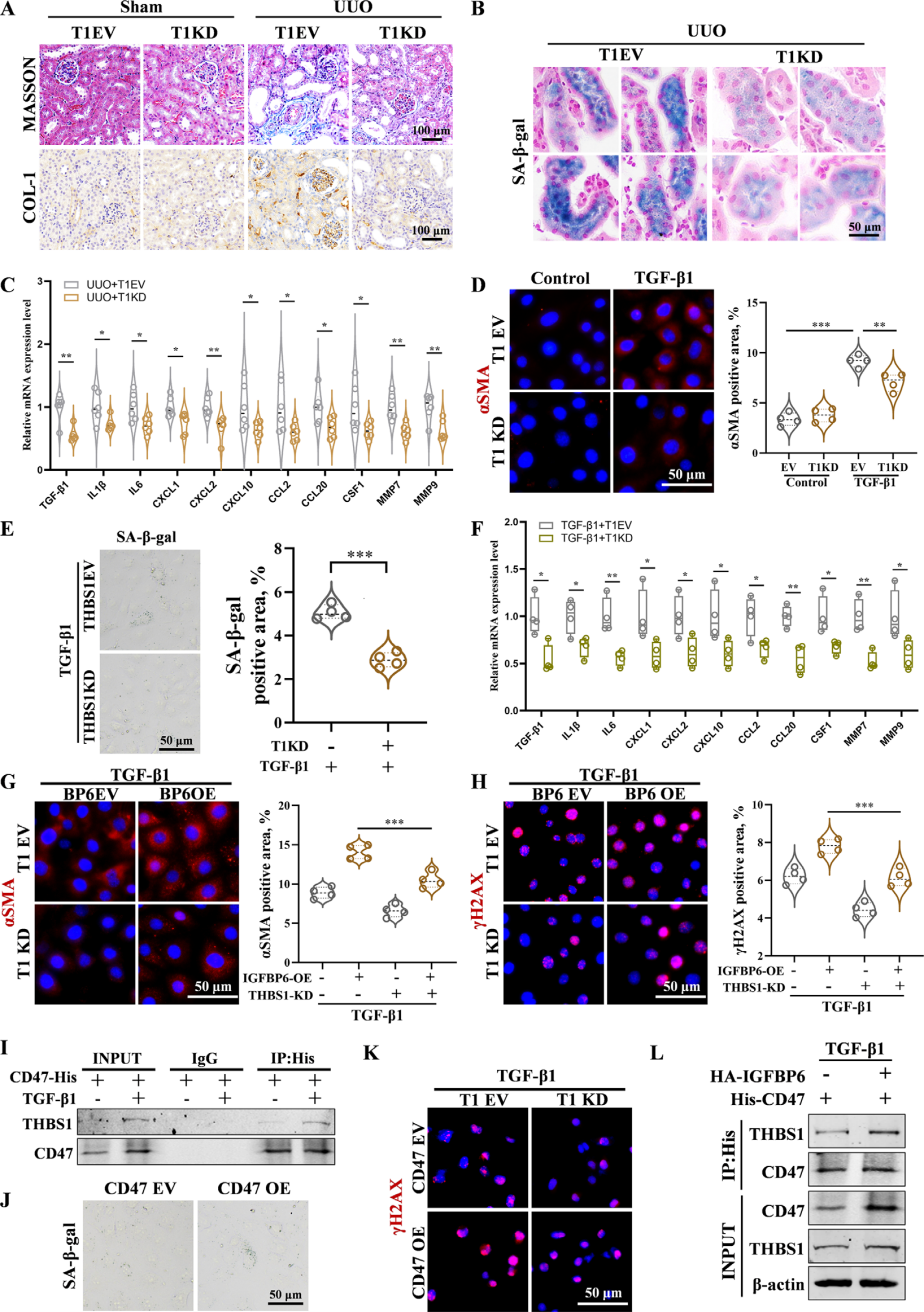
IGFBP6 promotes cellular senescence and renal fibrosis by promoting THBS1 binding to the receptor CD47. (A) The Masson staining and immunohistochemistry of COL‐1 in UUO mice model with THBS1 silencing (n = 30 views of 6 mice/group). (B) The SA‐β‐gal staining in UUO mice model with THBS1 silencing (n = 30 views of 6 mice/group). (C) The mRNA level of SASP‐related inflammatory factors in UUO mice model with THBS1 knockdown (n = 6 biological replicates). (D) Immunofluorescence of αSMA in TGF‐β1‐treated mTEC cells with THBS1 knockdown (n = 4). (E) The SA‐β‐gal staining in mTEC cells. (F) The mRNA level of SASP‐related inflammatory factors in mTEC cells with THBS1 silencing (n = 4 biological replicates). (G‐H) Immunofluorescence staining of αSMA and γH2AX after THBS1 knockdown in insulin‐like growth factor‐binding protein 6 (IGFBP6)‐OE mTEC cells treated with TGF‐β1 (n = 4 biological replicates, one‐way ANOVA). (I) Co‐immunoprecipitation (Co‐IP) assay detects the interactions of THBS1 and CD47 in TGF‐β1‐treated mTEC cells. (J) The SA‐β‐gal staining in mTEC cells with CD47 overexpression. (K) Immunofluorescence staining of γH2AX after THBS1‐KD in IGFBP6‐OE mTEC treated with TGF‐β1. (L) Co‐IP assay results detected interactions between THBS1 and CD47 in TGF‐β1‐treated mTEC cells overexpressing IGFBP6. Data are presented as mean ± SEM from at least 3–4 independent in vitro experiments and 6–8 mice in vivo. Statistical differences were determined using an independent sample t‐test and one‐way ANOVA with Tukey's post hoc analysis. *p < 0.05, **p < 0.01, ***p < 0.001.

Previous studies have shown a positive correlation between THBS1 expression in the kidney and the progression of renal fibrosis in patients with varying degrees of renal disease [30]. To investigate the functional role of THBS1, we conducted experiments involving THBS1 silencing and overexpression both in vivo and in vitro. Our in vivo studies, using Masson's trichrome staining, immunohistochemistry, SA‐β‐gal staining, and real‐time PCR, demonstrated that THBS1 silencing significantly reduced UUO‐induced renal fibrosis and senescence (Figure 6A‐C; Figure S9A,B). In vitro, we observed that THBS1 silencing markedly attenuated TGF‐β1‐induced cell fibrosis and senescence, while THBS1 overexpression significantly exacerbated these processes (Figure 6D‐F; Figure S9C,D). Notably, THBS1 secreted from renal tubular epithelial cells may modulate phenotypic transition and senescence of adjacent renal tubular epithelial cells through paracrine signaling (Figure S9F,G).

To further explore whether IGFBP6 regulates cellular senescence and fibrosis in HK2 cells through THBS1, we silenced THBS1 in IGFBP6‐overexpressing cells. The results demonstrated that THBS1 silencing alleviated the IGFBP6‐induced cellular senescence and fibrosis in TGF‐β1‐treated HK2 cells (Figure 6G,H; Figure S9E). CD47 and CD36 represent well‐characterized canonical receptors of THBS1, which have been reported to contribute to cell senescence [31, 32, 33]. However, our data revealed that silencing CD47 exerted a more pronounced inhibitory effect on the fibrotic‐like phenotype triggered by IGFBP6 overexpression relative to CD36 depletion (Figure S9H). Thus, CD47 was recognized as the principal downstream effector mediating IGFBP6‐THBS1 signaling. Notably, immunofluorescence co‑staining revealed prominent co‑localization of THBS1 and CD47 in fibrotic kidney tissues (Figure S9I). Co‐IP experiments further verified a direct physical interaction between THBS1 and CD47 in renal tubular epithelial cells (Figure 6I). We observed that CD47 overexpression significantly increased cellular senescence, as assessed by SA‐β‐gal staining (Figure 6J; Figure S9J). Overexpression of CD47 in cells with silenced THBS1 or IGFBP6 under TGF‐β1 stimulation restored the level of cell senescence (Figure 6K; Figure S9K,L). In addition, Co‐IP results indicated that IGFBP6 overexpression markedly enhanced the interaction between THBS1 and CD47 (Figure 6L). These findings collectively suggest that IGFBP6 promotes cell senescence by facilitating the interaction between THBS1 and CD47 during renal fibrosis.

### 2.7 Anti‐IGFBP6 Treatment Alleviates Renal Cellular Senescence and Fibrosis

**FIGURE 7:**
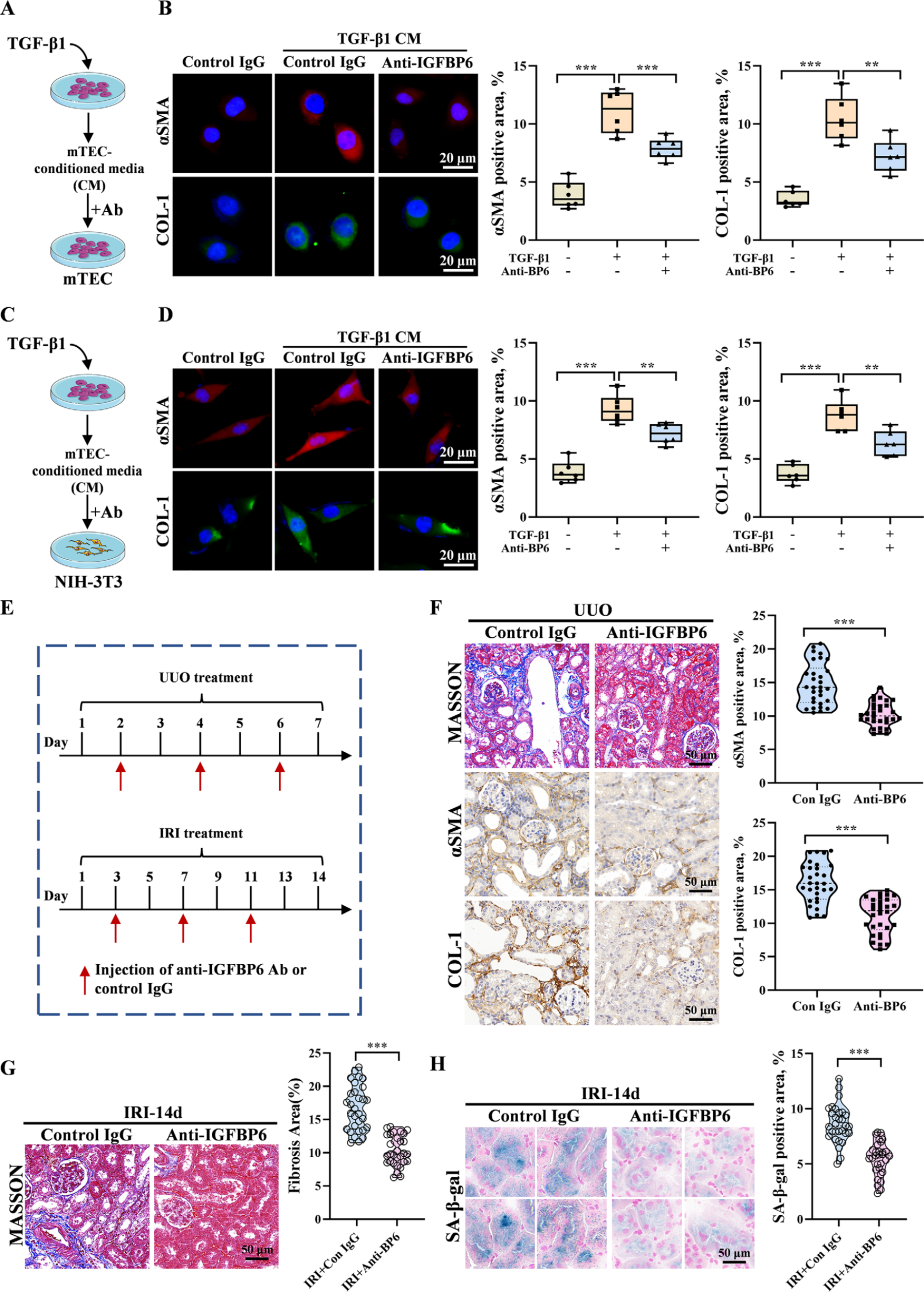
Anti‐IGFBP6 treatment can reduce the level of fibrosis and senescence in chronic kidney injury. (A) mTEC cells exposed to various conditioned media (CM) were administered anti‐IGFBP6 or control IgG. (B) Immunofluorescence staining of αSMA and COL‐1 in mTEC cells. (C) NIH‐3T3 cells stimulated with mTEC conditioned media with anti‐IGFBP6 or control IgG. (D) Immunofluorescence staining of αSMA and COL‐1 in NIH‐3T3 cells. (E) Scheme of the murine treatment and grouping in UUO and IRI‐induced renal fibrosis model. (F) Masson staining and immunohistochemical of αSMA and COL‐1 in UUO‐treated mice (n = 30 views of 6 mice/group). (G) Masson staining of IRI‐treated mice (n = 30 views of 6 mice/group). (H) The SA‐β‐gal staining in IRI‐induced renal fibrosis with or without anti‐IGFBP6 treatment (n = 30 views of 6 mice/group). Data are presented as mean ± SEM from at least 3–4 independent in vitro experiments and 6–8 mice in vivo. Statistical differences were determined using an independent sample t‐test and one‐way ANOVA with Tukey's post hoc analysis. **p < 0.01, ***p < 0.001.

Given the pathogenic role of IGFBP6 in promoting cellular senescence and kidney fibrosis, we explored targeting IGFBP6 as a therapeutic approach for renal fibrosis. We first investigated the therapeutic efficacy of IGFBP6‐neutralizing antibodies in epithelial cells and fibroblasts. In mTECs, immunofluorescence and Western blot analysis revealed that anti‐IGFBP6 antibody significantly reduced TGF‐β1‐induced α‐SMA and COL‐1 levels (Figure 7A,B; Figure S10A). Consistent with these findings, we demonstrated that anti‐IGFBP6 antibody attenuated TGF‐β1‐induced fibrosis in NIH‐3T3 cells (Figure 7C,D; Figure S10B). Encouraged by the positive effects of anti‐IGFBP6 antibody on reducing epithelial and fibroblast fibrosis in vitro, we further investigated its therapeutic potential in vivo.

More importantly, a monoclonal neutralizing antibody against IGFBP6 was also administered to UUO and IRI‐induced renal fibrosis mice (Figure 7E). Masson's trichrome staining, SA‐β‐gal staining, and immunohistochemistry revealed that anti‐IGFBP6 treatment significantly alleviated kidney injury, senescence, tubular dilatation, and fibrosis in UUO mice (Figure 7F; Figure S10C‐E). We also found that neutralizing antibody significantly reduced the level of fibrosis and senescence in the IRI induced renal fibrosis model (Figure 7G,H). Furthermore, the histological examination (hematoxylin and eosin staining) of vital organs, including the heart, liver, spleen, and lungs, revealed no discernible detrimental effects, confirming the lack of significant toxic side effects associated with Anti‐IGFBP6 administration within the tested dosage range (Figure S10F). Collectively, these data demonstrate the therapeutic potential of targeting IGFBP6 in treating renal fibrosis.

## 3 Discussion

Following injury, epithelial cells undergo dedifferentiation, apoptosis, and cellular senescence, accelerating the onset of renal fibrosis [34]. However, the underlying mechanisms remain poorly understood. While cell senescence is often associated with renal epithelial injury [35], its potential role in epithelial dedifferentiation and myofibroblast activation in chronic kidney disease is not well elucidated. In this study, we identified IGFBP6 as a key initiator of tubular damage, fibroblast activation, and cellular senescence. First, we demonstrated that tubular‐specific deletion of IGFBP6 significantly attenuated cell senescence and renal fibrosis through the inhibition of THBS1. Importantly, using a neutralizing antibody alleviated renal fibrosis in both cellular and animal models. Mechanistically, we found that epithelial IGFBP6 promotes renal fibrosis by enhancing CD47‐mediated cell senescence via THBS1‐dependent mechanisms. Our research indicates that IGFBP6 could be an effective therapeutic target for CKD patients (Figure S11).

Multiple lines of evidence have reported an association between IGFBP6 abundance and CKD, although the specific role of IGFBP6 in the process of kidney fibrosis remains unclear. We found that IGFBP6 was upregulated in fibrotic human kidney tissues and the UUO model, primarily localizing to tubular epithelium and, to a lesser extent, interstitial cells. These findings suggest that IGFBP6 may have specific functions in both tubular epithelial cells and interstitial fibroblasts in promoting renal fibrosis. Most studies support the concept that renal tubular epithelial cells are key drivers of renal fibrosis progression [36]. On the one hand, tubular epithelial cells are vulnerable to injury and may initiate maladaptive repair processes if the injury is irreversible [36]. On the other hand, tubular epithelial cells interact with other cell types through paracrine signaling, releasing various bioactive cytokines and inflammatory factors that can amplify inflammation and activate myofibroblasts [15, 37]. By utilizing renal tubular IGFBP6‐systemic and proximal tubular‐conditional knockout mice, we provided hitherto undocumented evidence of the role of IGFBP6 in mediating kidney fibrosis in UUO and IRI‐14d models. These results establish the central role of elevated IGFBP6 expression in tubular epithelial cells in driving kidney fibrosis progression. Mesenchymal fibroblasts are also considered a major source of extracellular matrix (ECM)‐producing myofibroblasts [38]. In vitro studies showed that overexpression of IGFBP6 in renal fibroblasts promoted ECM accumulation directly or indirectly by activating fibroblasts through conditioned medium from cultured tubular epithelial cells. Thus, our data indicate that IGFBP6 drives kidney fibrosis by activating both tubular epithelial cells and fibroblasts, acting as a cross‐talk mediator between these two cell types.

Chronic kidney disease is an irreversible medical condition with high morbidity, particularly among the elderly population. Renal fibrosis is identified as a consequence of accelerated cellular senescence within the kidney under pathological conditions [7, 39]. Senescent cells are known for a secretory phenotype that involves the secretion of proinflammatory and profibrotic cytokines. The promotion of renal fibrosis by these cytokines occurs through their effects on tubular cells, interstitial fibroblasts, and inflammatory cell infiltration. Recently, IGFBP6 was identified as a positive regulator of cellular senescence in diabetic kidney fibrosis, although the precise mechanism remains unclear [40]. Interestingly, our RNA‐seq analysis revealed that IGFBP6 significantly inhibited cell cycle and DNA repair pathways while promoting inflammation‐related signaling pathways, which are characteristic features of cellular senescence. Therefore, we investigated whether IGFBP6 could facilitate renal fibrosis by inducing cellular senescence. Our results demonstrated that IGFBP6 promoted the expression of cellular senescence markers (SA‐β‐gal and γH2AX) and SASP factors in renal tubular epithelial cells in vitro. Consistent with these findings, IGFBP6‐mediated cellular senescence and fibrogenic responses appear to operate within a self‐amplifying positive feedback loop: senescent cells secrete profibrotic SASP components to accelerate EMT and pathological ECM accumulation, while progressive fibrosis further primes and exacerbates cellular senescence in the renal parenchyma. The presence of p16INK4a‐positive cells in the kidney is a key biomarker for senescence and cancer research [41, 42]. Hironori Shimoda et al. found that p16INK4a accumulates in the IRI model, where TGF‐β1 induces p16INK4a expression through H3K4me3, ultimately contributing to renal fibrosis and inflammation [43]. Consistent with our in vitro findings, we also demonstrated that IGFBP6 conditional knockout significantly reduced UUO/IRI‐induced renal senescence markers (p16INK4a) and SASP accumulation. Overall, our findings indicate that IGFBP6 serves as an important trigger for cellular senescence in renal tubular epithelial cells, potentially contributing to renal fibrosis through this mechanism.

To better understand the precise mechanism by which IGFBP6 regulates cellular senescence and renal fibrosis, we employed LC‐MS/MS to screen for potential binding partners of IGFBP6. THBS1 emerged as a strong candidate, and Co‐IP results further confirmed a significant increase in the IGFBP6‐THBS1 interaction in UUO‐induced renal fibrosis and TGF‐β1‐stimulated HK2 cells. Notably, we found that IGFBP6 primarily binds to THBS1 through its TY domain. Moreover, we observed that increased IGFBP6 levels led to increased THBS1 expression. Ubiquitination and subsequent proteasomal degradation are essential for the efficient removal of proteins [44]. Using an E3 ubiquitin ligase prediction website, we identified NEDD4 as a potential E3 ligase for THBS1. Our study demonstrated that NEDD4 overexpression significantly increased THBS1 ubiquitination in HK2 cells. Crucially, we discovered that elevated levels of IGFBP6 led to a reduction in THBS1 ubiquitination and its interaction with NEDD4. A similar trend was observed in IGFBP6 knockdown HK2 cells. Collectively, our findings suggest that IGFBP6 primarily interacts with the TY region of THBS1, inhibiting its ubiquitination by counteracting the E3 ligase NEDD4.

To further investigate the functional role of THBS1 as a target of IGFBP6 in cellular senescence and renal fibrosis, we conducted both in vivo and in vitro studies. THBS1 is a member of the thrombin‐sensitive protein family, initially identified in platelets. It can form homotrimers and bind to cell surface receptors such as CD47, CD36, and lectin receptors. THBS1 has diverse biological functions, including anti‐angiogenesis, regulation of cell apoptosis, TGF‐β1 activation, and immune regulation, making it a key player in numerous cellular processes [27, 45]. The expression of THBS1 across various tissues, including the brain, muscle, lung, liver, and kidney, highlights its emerging role in the study of cellular senescence regulation. THBS1 has been implicated in several diseases, including metabolic disorders [46, 47] and chronic kidney diseases [30]. However, the specific role of THBS1 in promoting cellular senescence in kidney disease, particularly in kidney‐resident cells, remains incompletely understood. Our study demonstrated that silencing THBS1 both in vivo and in vitro significantly reduced cellular senescence and renal fibrosis. Importantly, THBS1 knockdown largely attenuated IGFBP6‐induced renal fibrotic responses, suggesting that IGFBP6 promotes renal fibrosis through a THBS1‐dependent mechanism. To gain insights into the role of THBS1 in regulating cellular senescence, we investigated its interaction with the CD47 receptor, a known suppressor of cell proliferation, as this pathway has been implicated in self‐renewal and reprogramming. Recent studies have shown that CD47‐high muscle stem cells (MuSCs) secrete high levels of THBS1, and THBS1/CD47 paracrine signaling suppresses aged MuSC proliferation [29]. Consistent with these findings, our study demonstrated that THBS1 binding to CD47 was significantly enhanced in TGF‐β1‐stimulated HK2 cells, and this interaction was further promoted by IGFBP6. Furthermore, we observed that overexpression of CD47 after silencing IGFBP6 or THBS1 could restore cellular senescence levels. Thus, our findings suggest that IGFBP6 promotes cellular senescence and renal fibrosis through a THBS1/CD47‐dependent mechanism.

It is worth noting that the pro‐fibrotic and pro‐aging effects of IGFBP6 in renal fibrosis may do not depend on the IGF‐2 signaling pathway. As a typical member of the IGFBP family, IGFBP6 is renowned for its high affinity for the key ligand of the classical IGF signaling pathway, IGF‐2 [48, 49]. However, our RNA sequencing analysis of renal tubular epithelial cells overexpressing IGFBP6 showed that the IGF‐related signaling pathways were not enriched, thus ruling out IGF‐2 as the key mediator in this process. Instead, IGFBP6 stabilizes THBS1 and activates the downstream THBS1‐CD47 signaling axis, which is the sole core pathway for its pro‐aging and pro‐fibrotic effects. Although IGFBP6 may regulate other biological processes through the IGF‐2 signal in different physiological or pathological environments, our data ultimately confirm that the renal cell aging and fibrosis driven by IGFBP6 mainly occurs through the THBS1‐CD47 signaling axis.

Given the significant therapeutic potential of targeting IGFBP6 in mitigating renal aging and fibrosis, we employed IGFBP6‐neutralizing antibodies in our therapeutic experiments. Despite advancements in our understanding of the mechanisms underlying kidney fibrosis, the global burden of CKD continues to rise, highlighting the urgent need for effective therapeutic interventions [21]. Our in vitro findings demonstrated that IGFBP6‐neutralizing antibodies significantly attenuated the fibrotic response in both TGF‐β1‐stimulated renal tubular epithelial cells and fibroblasts. Notably, we also observed that IGFBP6 neutralizing antibody significantly alleviated renal senescence and fibrosis in the UUO‐ and IRI‐induced renal fibrosis model.

Collectively, our findings identify IGFBP6 as a critical mediator of cellular senescence and renal fibrosis, correlated with diminished kidney function in chronic kidney disease. IGFBP6 is highly induced in TECs during renal fibrosis via an H3K4me3‐dependent mechanism. Mechanistically, IGFBP6 can antagonize the ubiquitination and degradation of THBS1 by inhibiting the E3 ubiquitin ligase NEDD4, leading to increased THBS1 expression and enhanced interaction between THBS1 and its receptor CD47, ultimately resulting in cellular senescence and fibrosis. Notably, neutralizing antibodies against IGFBP6 significantly mitigated senescence and fibrosis progression in both in vivo and in vitro models. In conclusion, our findings suggest that targeting IGFBP6 may provide a novel and effective therapeutic approach to impede the progression of renal fibrosis.

## 4 Materials and Methods

### 4.1 Study Design

The purpose of this research was to explore the function of IGFBP6 in renal fibrosis development and to clarify the mechanisms involved. To achieve this, we conducted a comprehensive study utilizing human serum, kidney tissues, various mouse models of CKD, and in vitro models of TGF‐β1‐induced renal tubular epithelial cells and fibroblasts. Patients with CKD, verified by estimated glomerular filtration rate, were recruited from the First Affiliated Hospital of Anhui Medical University. Kidney tissue without tumors from patients undergoing nephrectomy for renal cell carcinoma served as a normal control for analyzing IGFBP6 expression in kidney tissues. For our animal studies, we employed littermates whenever possible to ensure randomization. The sample size was determined based on ANOVA and our experience with similar studies. The sample size (n) for each experimental group, ranging from six to eight mice per group, is indicated in the figure legends. Within littermate groups, CKD was induced by UUO or IRI‐14d in mice with or without expression of IGFBP6 and THBS1. IGFBP6 neutralizing antibody was also administered to assess its therapeutic efficacy in UUO mice. In our cellular studies, at least three or four independent replicates were performed in tubular epithelial cells and fibroblasts with either knockdown or overexpression of IGFBP6 and THBS1. The figure legends indicate the number of replicates. Two independent pathologists, unaware of the sample identities, conducted all immunohistochemical quantifications. Statistical tests were selected based on the type of variables, assumptions about data distribution, and effect size.

### 4.2 Antibodies and Reagents

Antibodies used in this study included anti‐IGFBP6 (Proteintech, 67567‐1‐Ig; Abmart, PAQ4764), anti‐COL‐1 (Proteintech, 66761‐1‐Ig), anti‐αSMA (Proteintech, 14395‐1‐AP), anti‐VIM (ZENBIO, R22775), anti‐H3K4me3 (ABclonal, A22146), anti‐THBS1 (Proteintech, 18304‐1‐AP), anti‐CD47 (Proteintech, 20305‐1‐AP), anti‐NEDD4 (Proteintech, 21698‐1‐AP), anti‐γH2AX (ZENBIO, 381558), anti‐p16^INK4a^ (HUABIO, ET1608‐62), anti‐Flag (Proteintech, 20543‐1‐AP), and anti‐β‐actin (Servicebio, GB15001‐100). An IGFBP6‐neutralizing antibody was obtained from Sino Biological (50460‐R104). The IRDye 800‐conjugated secondary antibody was purchased from Li‐cor Biosciences (Nebraska, USA). Recombinant TGF‐β1 was purchased from PeproTech (New Jersey, USA), and recombinant IGFBP6 was purchased from R&D Systems (Minneapolis, MN, USA). MM‐102, OICR‐9429, and MG‐132 were purchased from APEXBio.

### 4.3 Human Samples

Samples of kidney tissue and peripheral blood were collected from both healthy individuals and chronic kidney disease patients at the First Affiliated Hospital of Anhui Medical University. Patients diagnosed with hydronephrosis via renal biopsy participated in the study. Normal controls were obtained from non‐tumor kidney tissue of patients who underwent nephrectomy for renal cell carcinoma. The serum of CKD patients was diagnosed by estimated glomerular filtration rate. Immediately after collection, the biopsy specimens were briefly rinsed with ice‐cold PBS to remove residual blood and debris, then snap‐frozen in liquid nitrogen within 5 min of isolation. The frozen samples were subsequently transferred to a −80°C ultra‐low temperature freezer for long‐term storage, and remained continuously frozen until protein extraction was performed. The clinical details for all donors are thoroughly outlined in Table S1. This study was approved by Biomedical Ethics Committee of Anhui Medical University (20190392). The studies were conducted with each participant's informed and written consent, following the guidelines of the Declaration of Helsinki.

### 4.4 Animal Studies

The animal experiments followed the Guide for the Care and Use of Laboratory Animals. Male C57BL/6J mice, aged 6 to 8 weeks and weighing approximately 20 to 22 grams, were acquired from the Experimental Animal Center at Anhui Medical University. All animal experiments were carried out at Anhui Medical University, with all procedures receiving approval from the Animal Experimentation Ethics Committee from Anhui Medical University, Anhui, China (20190443). For the UUO model, mice had their left ureter ligated for a span of 7 days. The IRI model was established by clipping bilateral renal pedicles in mice for 14 days. The Adenine model was induced by feeding mice a compound diet containing 0.2% adenine and 1.8% phosphorus (Xietong Biotechnology, Nanjing) as appropriate for 6 weeks. For AAV9‐mediated IGFBP6 overexpression in knockout mice, the adeno‐associated viruses were developed and obtained from HANBIO (Shanghai, China). For anti‐IGFBP6 treatment, neutralizing antibodies were injected intraperitoneally after UUO and IRI (Figure 7e). The control group of mice received an equivalent dose of rabbit IgG, and the treatment group of mice used a neutralizing IGFBP6 antibody (50 µg/mouse; Sino Biological, 50460‐R104). For further analysis, kidney tissues, blood, and urine were collected. Blood and urine were obtained for ELISA measurements as per the manufacturer's instructions. The kidneys were obtained for use in paraffin embedding, molecular analyses, and immunostaining experiments.

### 4.5 Generation and Verification of IGFBP6 Knockout Mice and Conditional Knockout Mice

IGFBP6 Global Knockout Mice: Global IGFBP6 knockout mice (C57BL/6) were generated by Cyagen Biosciences (Suzhou, China). Additionally, IGFBP6Flox/Flox mouse lines were produced by Cyagen Biosciences. IGFBP6 cKO mice were obtained by crossing IGFBP6 FF mice with Ggt1‐Cre mice, which possess a kidney‐specific promoter. Only male littermates aged between 8 and 10 weeks were used in the experiments, with PCR genotyping performed on all mice prior to experimentation.

### 4.6 Cell Culture Conditions, Transfection, and Treatments

The mTEC, HK2, NIH‐3T3, NRK‐52E, and NRK‐49F cells were cultured in 10% FBS‐containing HyClone DMEM/F12 medium in a humidified atmosphere of 5% CO_2_ at 37°C. Cells were cultured in HyClone DMEM. Plasmids were introduced into cells via lipofection. Cells were transfected with IGFBP6 siRNA, THBS1 siRNA, or a negative control, or with IGFBP6 and CD47 plasmids for 24 h. Following transfection, cells were starved overnight in a serum‐free medium and then treated with or without TGF‐β1 (10 ng/mL) for an additional 24 h, with 0.1% bovine serum albumin serving as a control. To assess the critical role of IGFBP6 in renal fibrosis, a neutralizing antibody was employed to evaluate its therapeutic potential. Conditioned medium was collected from mTEC cells pretreated with TGF‐β1 for 24 h (hereafter referred to as TGF‐β1‐treated mTEC cell‐conditioned medium). Similarly, conditioned medium was collected from mTEC cells pretreated with PBS (hereafter referred to as PBS‐treated mTEC cell‐conditioned medium). Fresh mTEC cells were divided into three groups. The three groups of mTEC cells were stimulated with (i) PBS‐treated mTEC cell‐conditioned medium and 25 µg/mL control IgG, (ii) TGF‐β1‐treated mTEC cell‐conditioned medium and 20 µg/mL control IgG, or (iii) TGF‐β1‐treated mTEC cell‐conditioned medium and 20 µg/mL IGFBP6‐neutralizing antibody (Sino Biological, 50460‐R104) for 24 h. In another experiment, the same three conditioned media were used to stimulate NIH‐3T3 cells. All cell lines underwent authentication through STR profiling, and Mycoplasma contamination was ruled out using the Myco‐Lumi Luminescent Mycoplasma Detection Kit for Low Sensitivity Instruments (Beyotime, China).

### 4.7 Western Blot

Following established protocols, protein lysates from kidney tissues and cultured cells were prepared, and western blot analyses were carried out as previously described [50]. Primary antibodies used in this study include IGFBP6, COL‐1, αSMA, VIM, H3K4me3, THBS1, CD47, NEDD4, Flag and β‐actin; IRDyeTM800‐conjugated secondary antibody (Rockland Immunochemicals). The LiCor/Odyssey infrared imaging system (LI‐COR Biosciences, Lincoln, NE, USA) was used to detect signals. The data were quantitatively assessed using Image J software (National Institutes of Health), and the relative expression was normalized against β‐actin.

### 4.8 RNA Isolation and Quantitative Real‐Time PCR

The RNeasy isolation kit (Qiagen, USA) was used to extract total RNA, which was then converted into cDNA with the reverse transcription kit (Bio‐Rad, Hercules, CA, USA). As previously detailed, real‐time PCR was conducted using the SYBR Green super mix and Opticon2 (Bio‐Rad, Hercules, CA) [51]. Quantitative RT‐PCR was conducted as described earlier [52]. The primer sequences employed in this research can be found in Table S2.

### 4.9 Overexpression and Knockdown of Genes

Plasmids or siRNA were transfected using Lipofectamine 2000 (Invitrogen, Carlsbad, CA) following the manufacturer's guidelines. After 6 h, the plasmids were changed into fresh with medium, followed by analyses 48–72 h later. For AAV(Ggt1)‐mediated IGFBP6 OE in IGFBP6‐KO mice, the adeno associated viruses were developed and obtained from GeneChem (Shanghai, China). The AAV‐packaged IGFBP6‐OE plasmid (1 × 10^12^ vg/mL) was injected into the renal pelvis of mice. For renal pelvic injection, mice were anesthetized and the kidney exposed through a flank incision. The renal pelvis was exposed and AAV particles injected into it using a 31‐gauge needle. For lentivirus‐mediated THBS1 KD in mice, lentiviruses with THBS1 KD and empty vector plasmids were purchased from GenePharma (Shanghai, China).

### 4.10 Immunostaining

Kidney tissue sections, three to five micrometers thick, were fixed in formalin, embedded in paraffin, deparaffinized, rehydrated, and stained using Masson staining, as previously outlined [16]. The IHC staining procedure followed the guidelines provided by the manufacturer. Deparaffinized sections were incubated with primary antibodies against IGFBP6 (Proteintech, 67567‐1‐Ig), COL‐1 (Proteintech, 66761‐1‐Ig), α‐SMA (Proteintech, 14395‐1‐AP), and p16^INK4a^ (HUABIO, ET1608‐62) overnight at 4°C, followed by incubation with secondary antibodies for 30 min at room temperature. After counterstaining with DAB, the sections were examined under a microscope (Leica, Germany).

For immunofluorescence staining, cells were cultured on six‐well glass slides, fixed with acetone, and blocked with 5% goat serum in PBS for 1 h at room temperature. Subsequently, cells were incubated overnight at 4°C with primary antibodies against IGFBP6 (Proteintech, 67567‐1‐Ig), αSMA (Proteintech, 14395‐1‐AP; Proteintech, 67735‐1‐Ig), COL‐1 (Proteintech, 14695‐1‐AP), γH2AX (ZENBIO, 381558), or VIM (ZENBIO, R22775). Following PBS washing, the cells were treated with secondary antibodies and DAPI stain at room temperature. In order to visualize the brush border of the proximal tubule, kidney sections were stained using fluorescein isothiocyanate (FITC)‐labeled Lotus tetragonolobus lectin (LTL; Vector Laboratories, USA). Following dewaxing, tissue sections or tissue chips were subjected to either EDTA or citrate antigen retrieval in a microwave oven, depending on the antibody requirements. Deparaffinized sections were incubated overnight at 4°C with primary antibodies against IGFBP6 (Bioss, bs‐4064R), α‐SMA (Proteintech, 14395‐1‐AP), or VIM (ZENBIO, R22775). The samples were subsequently exposed to a secondary antibody, and images were captured and viewed using a microscope.

### 4.11 ELISA for Albumin in Urine and IGFBP6

The levels of IGFBP6 in serum and cell culture supernatants were determined by human, rat, or mice IGFBP6 ELISA kit (Aimeng Youning, Shanghai, China). The procedures outlined by the manufacturer were used for the assays.

### 4.12 Immunoprecipitation Coupled with Mass Spectrometry (IP‐MS/MS)

Identification of IGFBP6 interacting proteins by IP‐MS/MS analysis. HK2 cells were overexpressed with Flag‐IGFBP6. Proteins were separated using SDS‐PAGE, then reduced, alkylated, and digested with trypsin in situ. Proteins were obtained from transfected cells, and immunoprecipitation was conducted with primary antibodies targeting protein A/G‐agarose beads. The collected immunoprecipitates were examined using mass spectrometry (Shanghai Luming biological technology, China).

### 4.13 ChIP Assay

The anti‐H3K4me3 antibody and normal IgG were used for immunoprecipitation, with incubation taking place overnight at 4°C. SimpleChIP Enzymatic Chromatin IP Kit (#9003, Cell Signaling Technology, USA) was used as described previously. The analysis of the IGFBP6 promoter region utilized the 7500 Fast Real‐Time PCR System (Applied Biosystems), with SYBR green Premix Ex Taq (Takara Bio, Kusatsu, Japan) and the following pairs of human IGFBP6 primers: forward, 5'‐ TTTTGCCCTTCCTCTCCTCT‐3', and reverse, 5'‐ ACGGGAGTGCAATCCACTTA ‐3'.

### 4.14 SA‐β‐Gal Staining

Soak and wash the tissue in PBS for three times, each time for 5 min. Add β‐galactosidase staining fixative, cover the tissue thoroughly, and fix at room temperature for 15 min. After three additional 5‑min washes in PBS, an appropriate volume of staining solution was added, and tissues were incubated overnight at 37°C. Following incubation, sections were counterstained with nuclear fast red to enhance nuclear contrast and facilitate the visualization of SA‐β‐gal positive signals. Stained sections were observed and imaged under a conventional light microscope.

### 4.15 Co‐Immunoprecipitation

Co‐IP analysis was executed according to prior descriptions [53, 54]. In summary, the cells were rinsed three times with ice‐cold PBS solution and then lysed using a 10% NP‐40 buffer. To summarize, cells underwent three washes with ice‐cold PBS solution and were lysed in a 10% NP‐40 buffer. The samples were precipitated with control IgG antibody, primary antibody, and protein A/G–agarose beads (Bio‐linkedin, China) by being incubated overnight at 4°C. Bound proteins were eliminated by boiling in SDS buffer and then separated on 4% to 20% SDS–polyacrylamide gels for Western blot analysis with Flag, His, THBS1, and NEDD4 antibody.

### 4.16 Statistical Analyses

Normalization of data was achieved by dividing the measured values by the corresponding internal control values, yielding a new value. Then, the average for the control or sham group was computed. Finally, the normalized data were produced by dividing each group's new values by this mean. All statistical analyses were performed using SPSS version 23.0 (SPSS Inc., Chicago, IL, USA). Figures were created using GraphPad Prism 9.0 from GraphPad Software Inc. All results were assessed for normality using the Shapiro‐Wilk test. Quantitative data that followed a normal distribution are presented as means ± SEM. Differences in group means were analyzed using independent sample t‐tests and one‐way ANOVA, followed by Tukey's post hoc test. A *p*‐value < 0.05 was considered statistically significant.

## Author Contributions

X‐M.M., W.W., and J‐T.Y. supervised the research, designed the experiments, and wrote the paper. J‐T.Y., C.‐G.X., X‐W.H., and R‐R.S. performed most of the experiments. J‐T.Y., J‐N.W., X‐Y.L., M‐M.Z., X‐Y.S., Y‐Q.W., and J‐M.Y. performed in vitro studies. J‐T.Y., R.H., Y‐H.D., S.S., C.L., and C.H. performed in vivo studies. J.W., Z‐J.W., D‐F.Z., T.Z., and Y‐X.R. performed histological analyses. J‐T.Y., J.J., J‐G.W., Y‐J.L., and M‐M.L. interpreted the results. W.W., X‐M.M., J‐T.Y., and X‐W.H. acquired funding to support the project. W.W., J‐T.Y., and X‐M.M. wrote the manuscript. All authors read and approved the final manuscript.

## Funding

This work was supported by the National Natural Science Foundation of China (No. 81900616, 82270737, 82500846, 82470732), Natural Science Foundation of Anhui Province (No. 2508085QH348), Outstanding Youth Project of Natural Science Foundation for Universities in Anhui Province (2023AH020047), and Scientific Research Project of Education Department of Anhui Province (China, No. 2025AHGXZK30106, 2023AH053398).

## Conflicts of Interest

None of the authors have a conflict of interest to disclose.

## Supporting information




**Supporting File**: advs75802‐sup‐0001‐SuppMat.pdf.

## Data Availability

The data that support the findings of this study are available from the corresponding author upon reasonable request. The data that support the findings of this study are openly available in NCBI Sequence Read Archive (SRA) at https://www.ncbi.nlm.nih.gov/bioproject/PRJNA1218417, reference number PRJNA1218417.
