# Supplementary material for: Renal IGFBP6 Interacts With THBS1 to Drive Renal Cellular Senescence and Fibrosis
Source: Adv Sci (Weinh). 2026 May 22:e75802. Online ahead of print. doi: 10.1002/advs.75802 (PMC13335767; doi:10.1002/advs.75802)
Supplement: Supplementary file 1 — Supporting File: advs75802‐sup‐0001‐SuppMat.pdf. [file ADVS-9999-e75802-s001.pdf]

Supplementary information

Figure S1. IGFBP6 expression is greatly elevated in human biopsies, mouse models, mTEC and NRK-49F cells in response to CKD.

Figure S2. H3K4me3 activation in CKD leads to increased expression of IGFBP6.

Figure S3. IGFBP6 deletion protects against UUO/IRI-14d-induced renal fibrosis.

Figure S4. IGFBP6 conditional knockout and restore experiment.

Figure S5. IGFBP6 promotes fibrotic response of renal tubular epithelial cells.

Figure S6. IGFBP6 promotes fibrosis in fibroblasts cells.

Figure S7. IGFBP6 promote cell senescence.

Figure S8. IGFBP6 binds to THBS1 and antagonizes its ubiquitination degradation by E3 ubiquitin ligase NEDD4.

Figure S9. IGFBP6 promotes the binding of THBS1 and its receptor CD47, leading to the senescence of renal tubular epithelial cells.

Figure S10. Anti-IGFBP6 treatment can reduce the level of fibrosis and senescence in chronic kidney injury.

Figure S11. IGFBP6 promotes renal fibrosis by enhancing THBS1/CD47-mediated cell senescence.

Table S1. Clinical features of patients with chronic kidney disease in this study.

Table S2. Primers and RNA sequences used in this study.

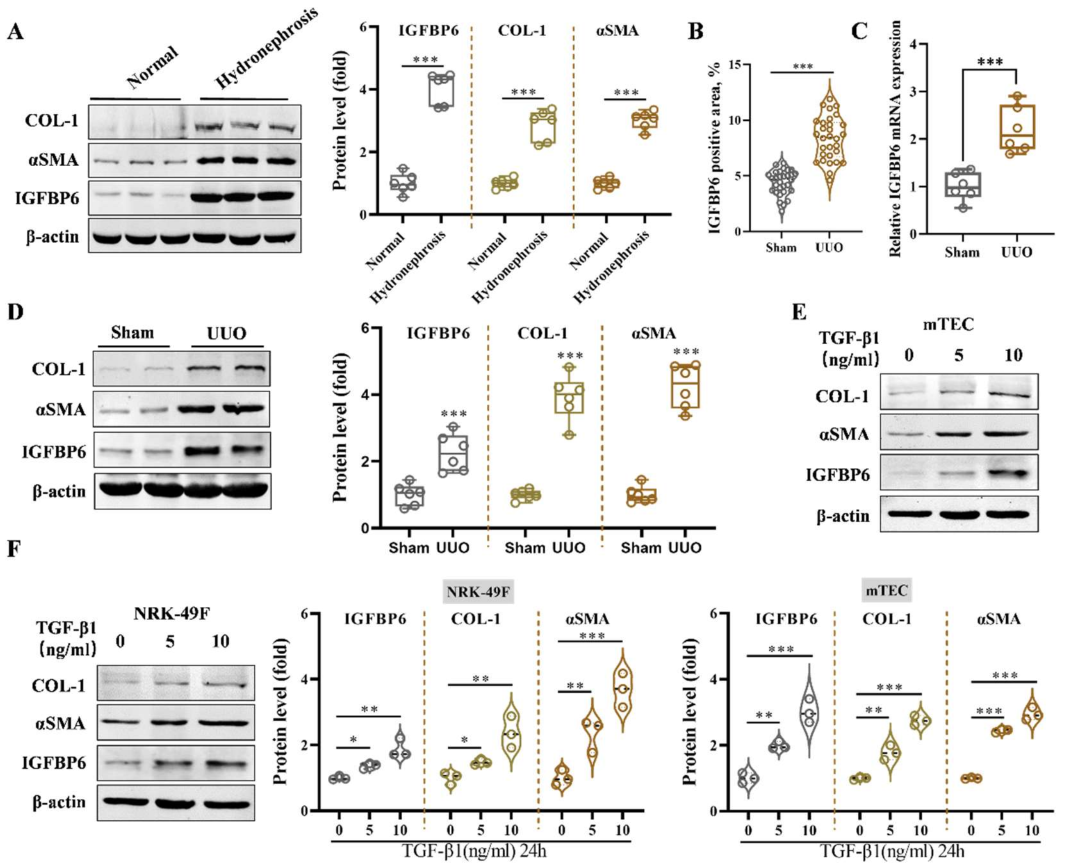

Figure S1. IGFBP6 expression is greatly elevated in human biopsies, mouse models, mTEC and NRK-49F cells in response to CKD.

(A) Protein level of COL-1, αSMA and IGFBP6 in human biopsy from CKD patients (normal: n=6; hydronephrosis: n=6). (B) Representative immunofluorescence staining quantitative of IGFBP6 in UUO mice model (n=30 views of 6 mice/group). (C) mRNA level of IGFBP6 in UUO mice model (n=6 mice/group). (D) Protein level of COL-1, αSMA and IGFBP6 in UUO mice model (n=6 mice/group). (E-F) Protein level of COL-1, αSMA and IGFBP6 in TGF-β1-induced mTEC and NRK-49F cells model. Data are presented as mean ± SEM from at least 3-4 independent *in vitro* experiments and 6-8 mice *in vivo*. Statistical differences were determined using an independent sample t-test and one-way ANOVA with Tukey's post hoc analysis. **P < 0.01, ***P < 0.001.

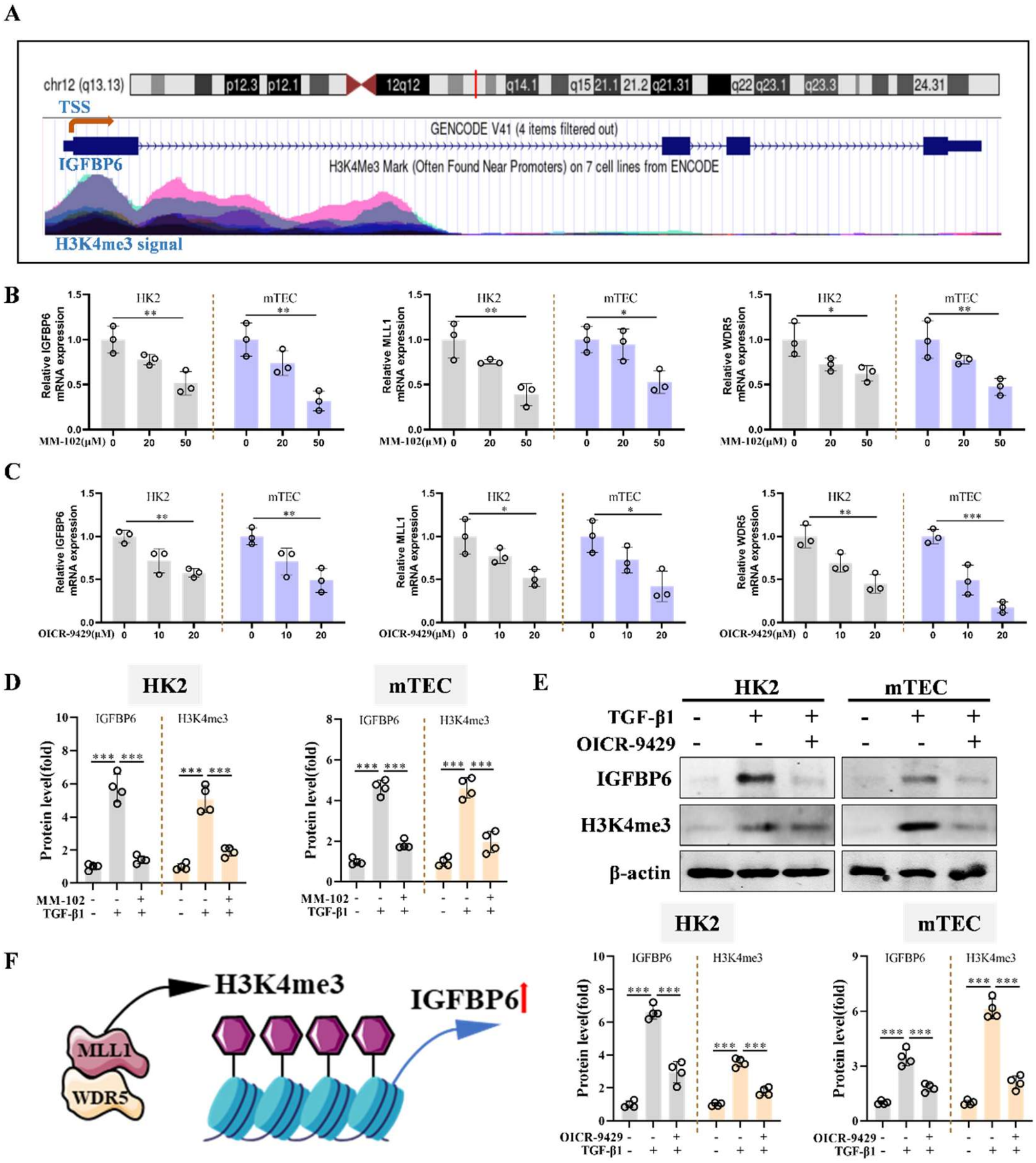

Figure S2. H3K4me3 activation in CKD leads to increased expression of IGFBP6.

(A) The UCSC genome bioinformatics site data indicated a significant enrichment of H3K4me3 in the IGFBP6 promoter (http://genome.ucsc.edu/). (B) Real-time PCR analyses of IGFBP6, MLL1 and WDR5 in TEC cells with MM-102 stimulation. (C) Real-time PCR analyses of IGFBP6, MLL1 and WDR5 in TEC cells with OICR-9429 stimulation. (D) Western blot quantitative analyses of H3K4me3 and IGFBP6 in TEC cells with MM-102 stimulation. (E) Western blot analyses of H3K4me3 and IGFBP6

in TEC cells with OICR-9429 stimulation. (F) Graphical illustration of the mechanism by which MLL1/WDR5-mediated H3K4me3 activation promotes IGFBP6 transcription. Data are presented as mean ± SEM from at least 3-4 independent *in vitro* experiments. Statistical differences were determined using an independent sample t-test and one-way ANOVA with Tukey's post hoc analysis. $*P < 0.05$, $**P < 0.01$, $***P < 0.001$.

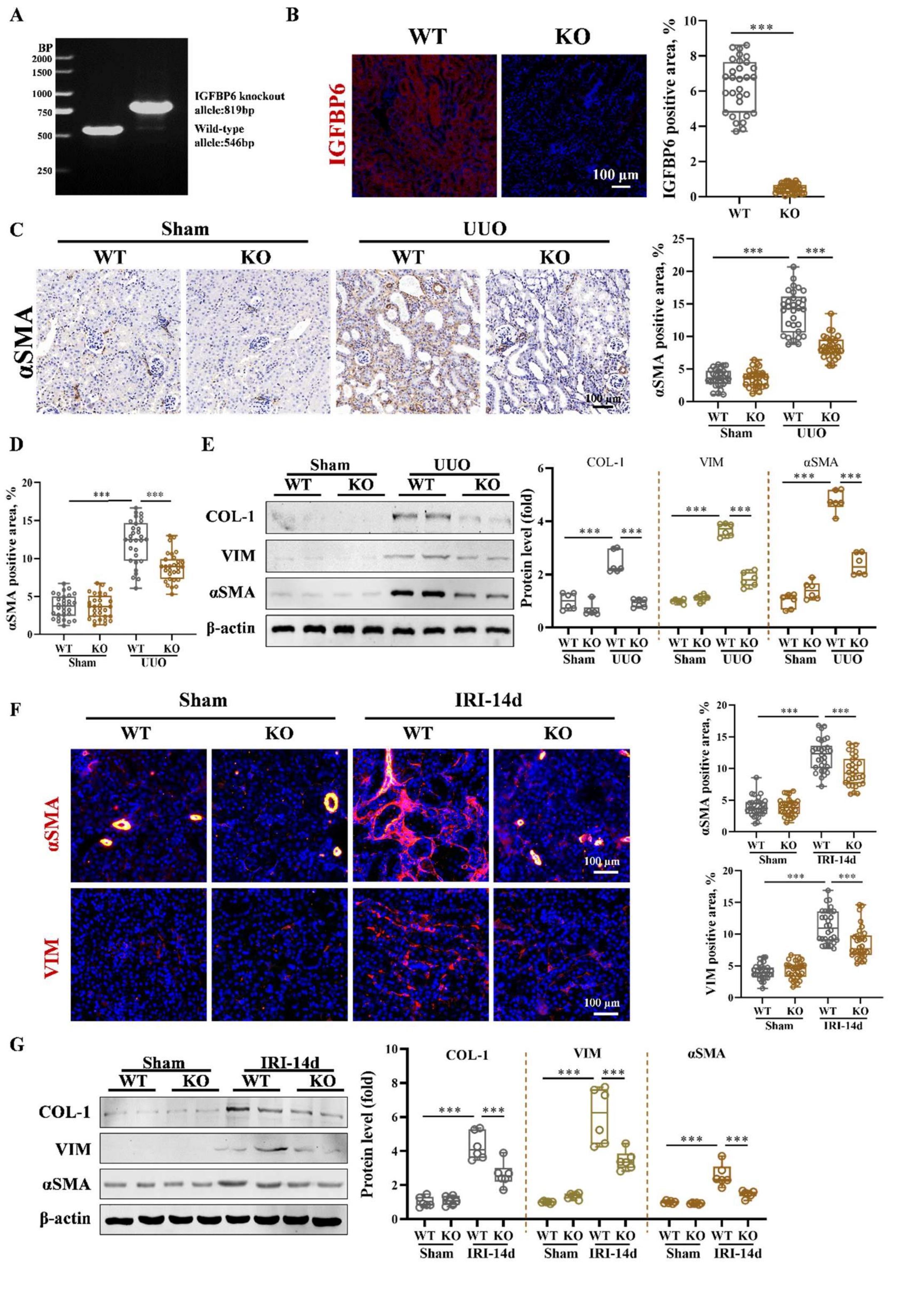

Figure S3. IGFBP6 deletion protects against UUO/IRI-14d-induced renal fibrosis.

(A) The PCR assay for mice genotype confirmed IGFBP6 deficiency by detecting genomic DNA. (B) Representative IF staining of IGFBP6 in IGFBP6-KO mice compared with WT mice (n=30 views of 6 mice/group). (C) Immunohistochemistry

staining of αSMA in UUO-treated IGFBP6-KO mice (n=30 views/group). (D) Quantitative results of immunofluorescence for αSMA in kidney from WT and IGFBP6-KO mice treated with UUO (n=30 views of 6 mice/group). (E) Western blotting analyses of COL-1, αSMA, and VIM expression in IGFBP6-KO mice compared with control (n = 6 mice/group). (F)Immunofluorescence staining of αSMA and VIM in IRI-14d-treated IGFBP6-KO mice (n=30 views/group). (G) Western blotting analyses of COL-1, αSMA and VIM expression in IGFBP6-KO mice compared with IRI-treated IGFBP6-KO mice (n=6 mice/group). Data are presented as mean ± SEM from at least 6-8 mice *in vivo*. Statistical differences were determined using an independent sample t-test and one-way ANOVA with Tukey's post hoc analysis. ***P < 0.001.

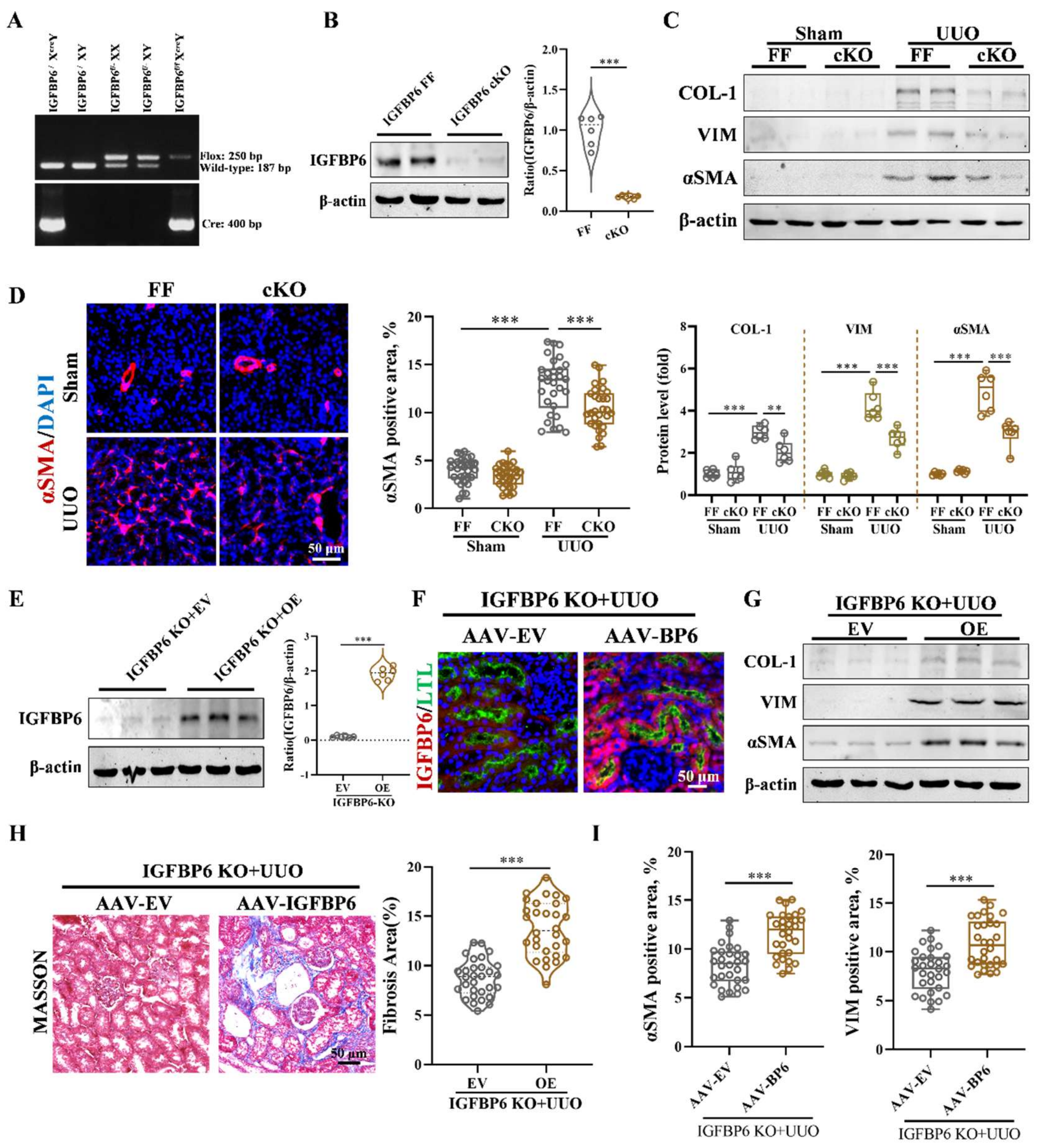

Figure S4. IGFBP6 conditional knockout and restore experiment.

(A) Genotype PCR assay of IGFBP6 tubular conditional knockout mice. (B)Western blotting analyses of IGFBP6 in renal cortical lysates from IGFBP6-cKO mice (n=6 mice/group). (C) Western blotting analyses of COL-1, αSMA and VIM expression in IGFBP6-cKO mice compared with control (n=6 mice/group). (D) Immunofluorescence staining quantitative of αSMA in UUO-treated IGFBP6-cKO mice (n = 30 views/group). (E) The western blotting results analyses of IGFBP6 (n=6 mice/group). (F) Representative immunofluorescence staining of IGFBP6 and LTL in UUO-treated mice

with IGFBP6 overexpression. (G) Western blotting analyses of COL-1, αSMA and VIM expression (n=6 mice/group). (H) Representative Masson staining pictures of kidneys from IGFBP6 KO mice with IGFBP6 overexpression (n = 30 views of 6 mice/group). (I) Relative quantitative analysis of immunofluorescence staining and quantification of αSMA and VIM in KO mice with IGFBP6 overexpression (n=30 views of 6 mice/group). Data are presented as mean ± SEM from at least 6-8 mice *in vivo*. Statistical differences were determined using an independent sample t-test and one-way ANOVA with Tukey's post hoc analysis. **P < 0.01, ***P < 0.001.

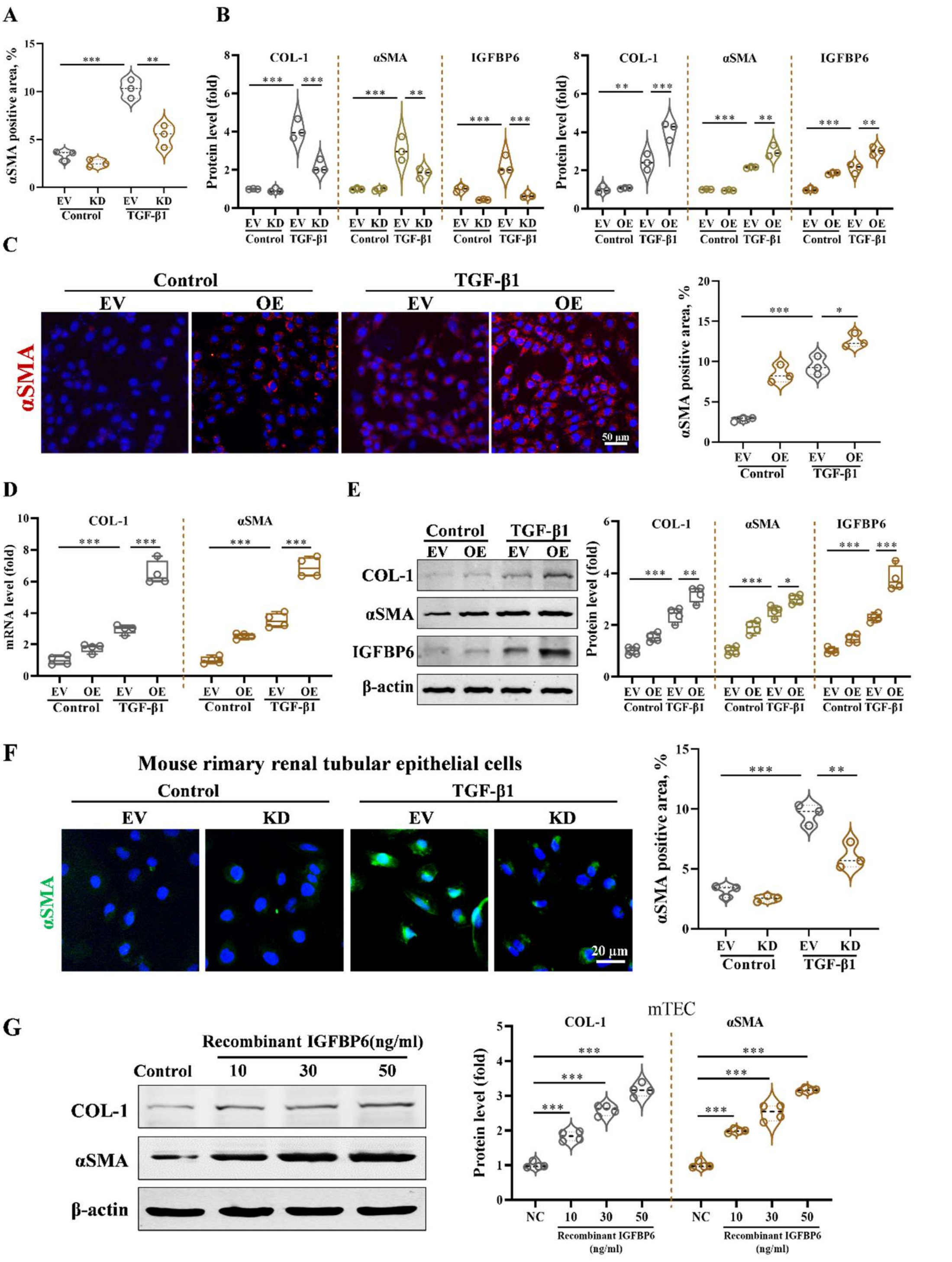

Figure S5. IGFBP6 promotes fibrotic response of renal tubular epithelial cells.

(A) Immunofluorescence quantification of αSMA with silencing IGFBP6 in mTEC cells. (B) Western blot quantification analyses of COL-1, αSMA and IGFBP6. (C) IF staining of αSMA. (D) Real-time PCR of COL-1 and αSMA. (E) Western blot and

quantification analyses of COL-1, αSMA and IGFBP6 in NRK-52E cells. (F) Immunofluorescence quantification of αSMA with silencing IGFBP6 in primary renal tubular epithelial cells. (G) Recombinant protein stimulation induces mTEC fibrosis levels. Western blot and quantification analyses of COL-1 and αSMA in mTEC cells. Data are presented as mean ± SEM from at least 3-4 independent *in vitro* experiments. Statistical differences were determined using an independent sample t-test and one-way ANOVA with Tukey's post hoc analysis. *$P < 0.05$, **$P < 0.01$, ***$P < 0.001$.

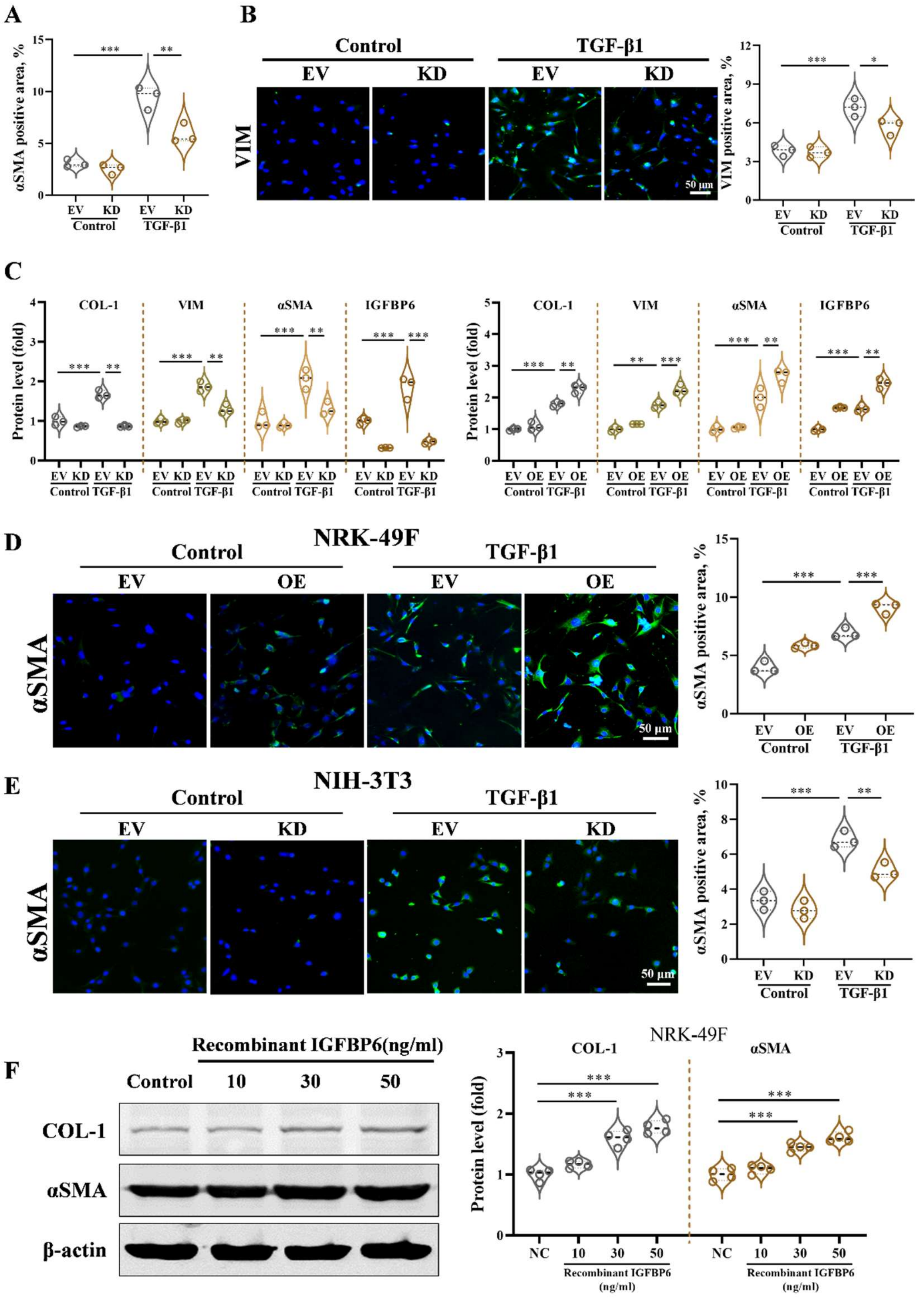

Figure S6. IGFBP6 promotes fibrosis in fibroblasts cells.

(A) Quantitative analyses for IF of αSMA with silencing IGFBP6 in NRK-49F cells. (B) Immunofluorescence staining of VIM. (C) Quantitative analyses for WB of COL-1, VIM, αSMA and IGFBP6. (D) Immunofluorescence staining of αSMA in NRK-49F

cells. (E) Immunofluorescence staining of αSMA in NIH-3T3 cells. (F) Recombinant protein stimulation induces NRK-49F fibrosis levels. Western blot and quantification analyses of COL-1 and αSMA in NRK-49F cells. Data are presented as mean ± SEM from at least 3 independent *in vitro* experiments. Statistical differences were determined using an independent sample t-test and one-way ANOVA with Tukey's post hoc analysis. $^{*}P < 0.05$, $^{**}P < 0.01$, $^{***}P < 0.001$.

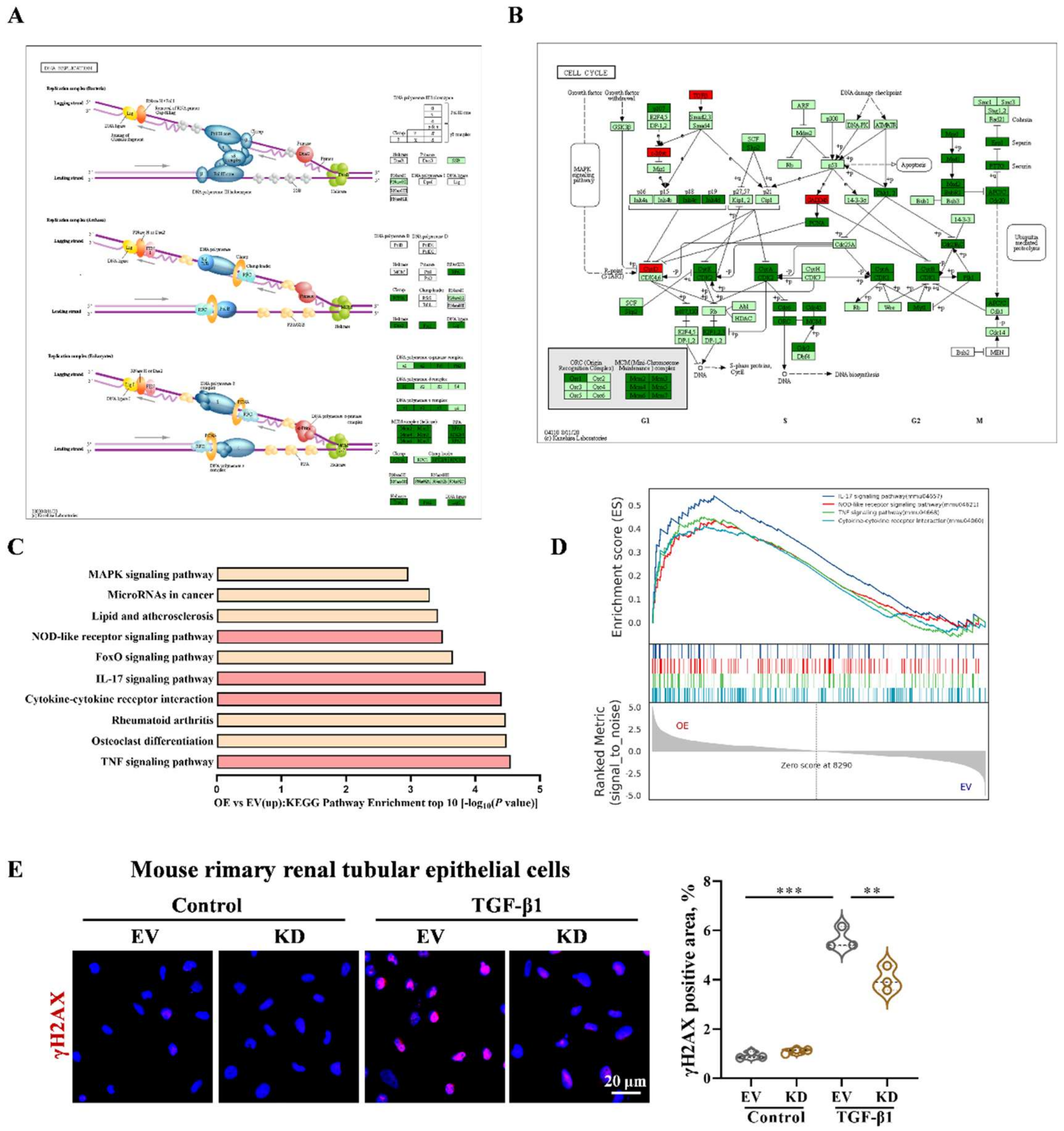

Figure S7. IGFBP6 promote cell senescence.

(A) KEGG map of DNA replication pathway. (B) KEGG map of Cell cycle pathway. (C) Up-regulated gene KEGG pathway enrichment in mTEC cells with IGFBP6 overexpress. (D) The GSEA analysis of genes with altered expression in mTEC cells overexpressing IGFBP6 reveals a significant enrichment in the pathway related to SASP inflammatory factors. (E) Immunofluorescence quantification of γH2AX with silencing IGFBP6 in primary renal tubular epithelial cells. Data are presented as mean ± SEM from at least 3-4 independent *in vitro* experiments. Statistical differences were

determined using an independent sample t-test and one-way ANOVA with Tukey's post hoc analysis.

A

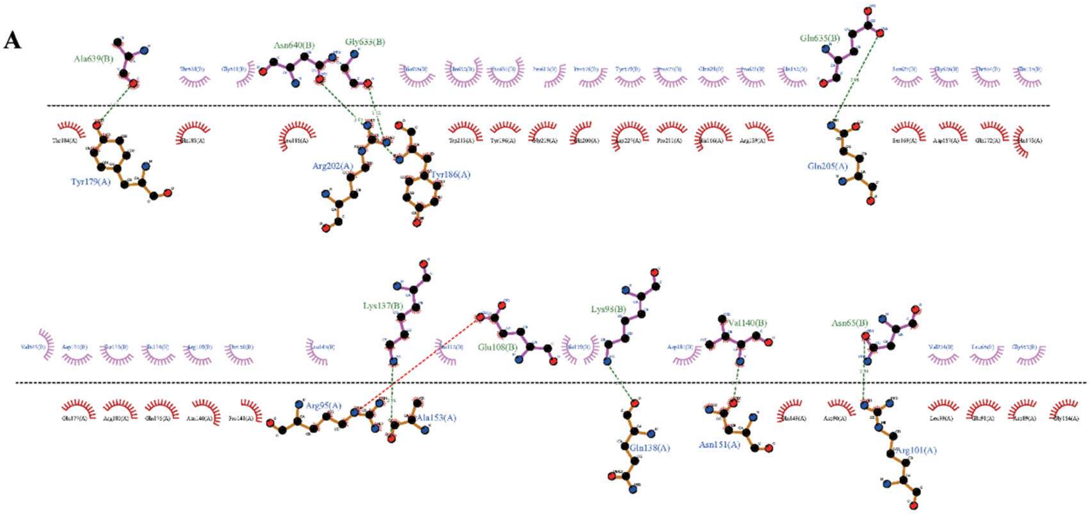

B **Known ubiquitination sites and predicted recognizing motif/domain for substrate: THBS1 mediated by E3 ligase: NEDD4**

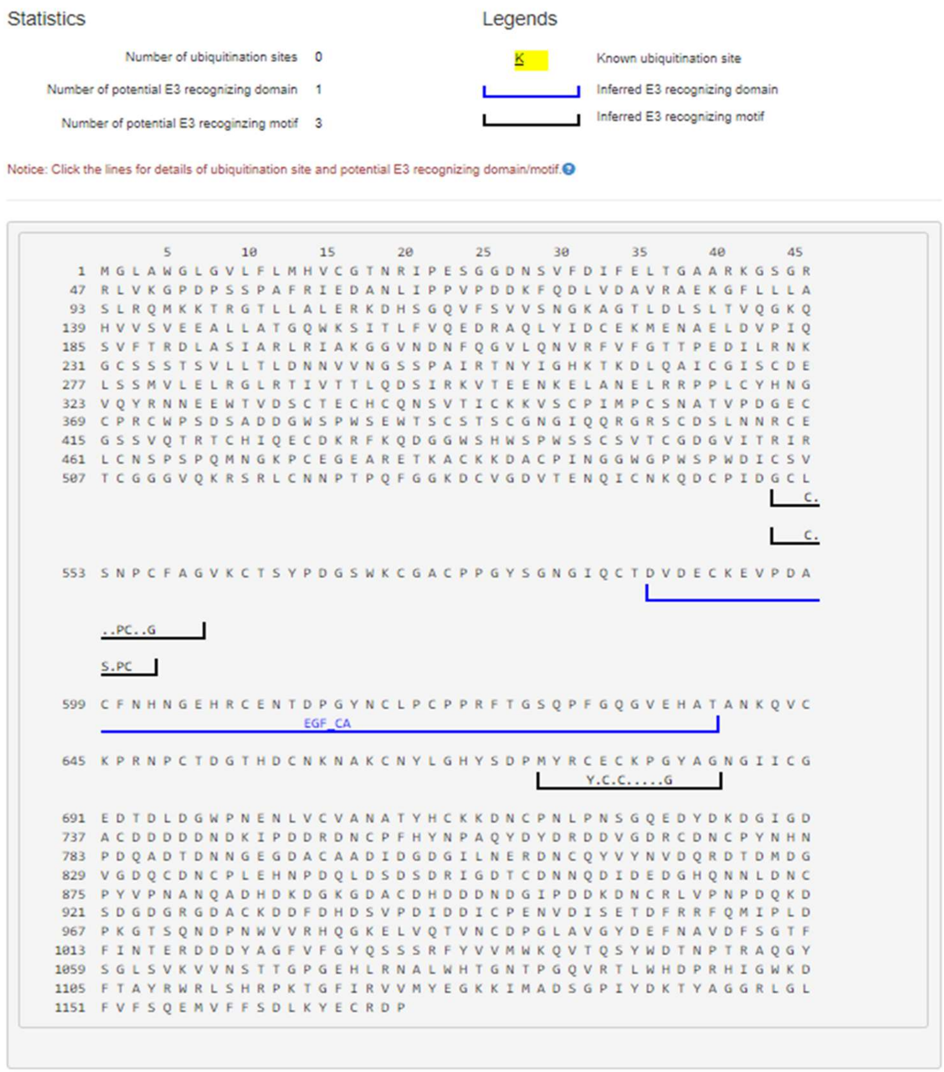

Figure S8. IGFBP6 binds to THBS1 and antagonizes its ubiquitination

degradation by E3 ubiquitin ligase NEDD4.

(A) Speculation of binding sites between IGFBP6 and THBS1. (B) NEDD4 binding site to THBS1. Known ubiquitination sites and predicted recognizing motif/domain for substrate: THBS1 mediated by E3 ligase: NEDD4.

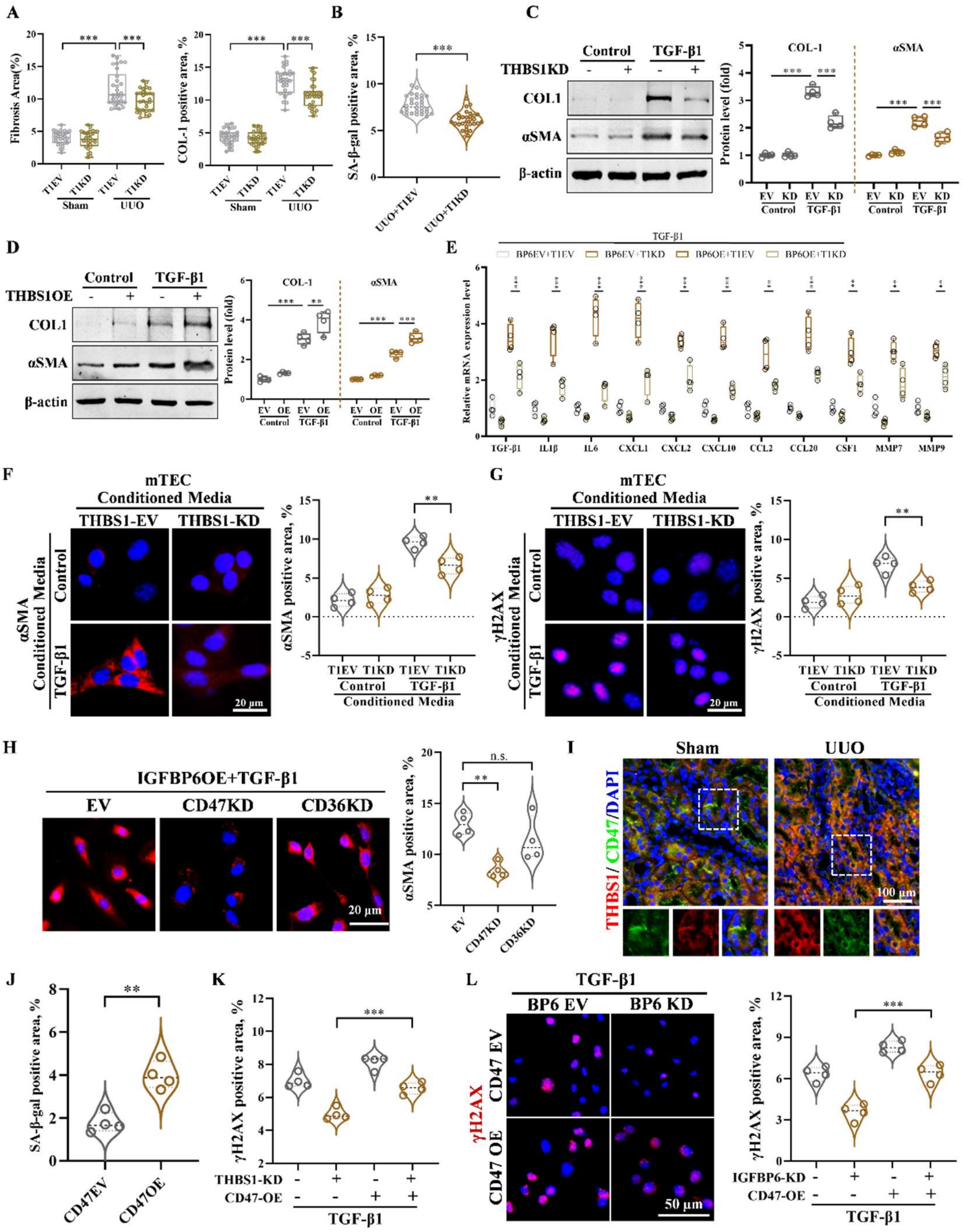

Figure S9. IGFBP6 promotes the binding of THBS1 and its receptor CD47, leading to the senescence of renal tubular epithelial cells

(A) Quantitative analyses of Masson staining and immunohistochemistry of COL-1 in UUO mice model with THBS1 silencing (n = 30 views of 6 mice/group). (B) Quantitative analyses of SA-β-gal staining in UUO mice model with THBS1 silencing

(n = 30 views of 6 mice/group). (C-D) Western blot analyses of COL-1 and αSMA in TGF-β1-treated mTEC cells with THBS1 silencing/overexpression. (E) The mRNA level of SASP-related inflammatory factors in IGFBP6-OE mTEC with THBS1 knockdown (n = 4 biological replicates). (F-G) Immunofluorescence staining of αSMA and γH2AX in mTEC. (H) IF staining of αSMA and γH2AX in IGFBP6 OE mTEC with CD47 or CD36 knockdown. (I) Representative immunofluorescence staining of THBS1 and CD47 in UUO mice model. (J) Quantitative analyses of SA-β-gal staining in mTEC cells with CD47 overexpression. (K) Quantitative analyses of immunofluorescence staining of γH2AX in THBS1-KD TGF-β1-treated mTEC with CD47 overexpression. (L) Immunofluorescence staining of γH2AX after IGFBP6-KD in CD47-OE mTEC treated with TGF-β1. Data are presented as mean ± SEM from at least 3 independent *in vitro* experiments and 6 mice *in vivo*. Statistical differences were determined using an independent sample t-test and one-way ANOVA with Tukey's post hoc analysis. **$P < 0.01$, ***$P < 0.001$.

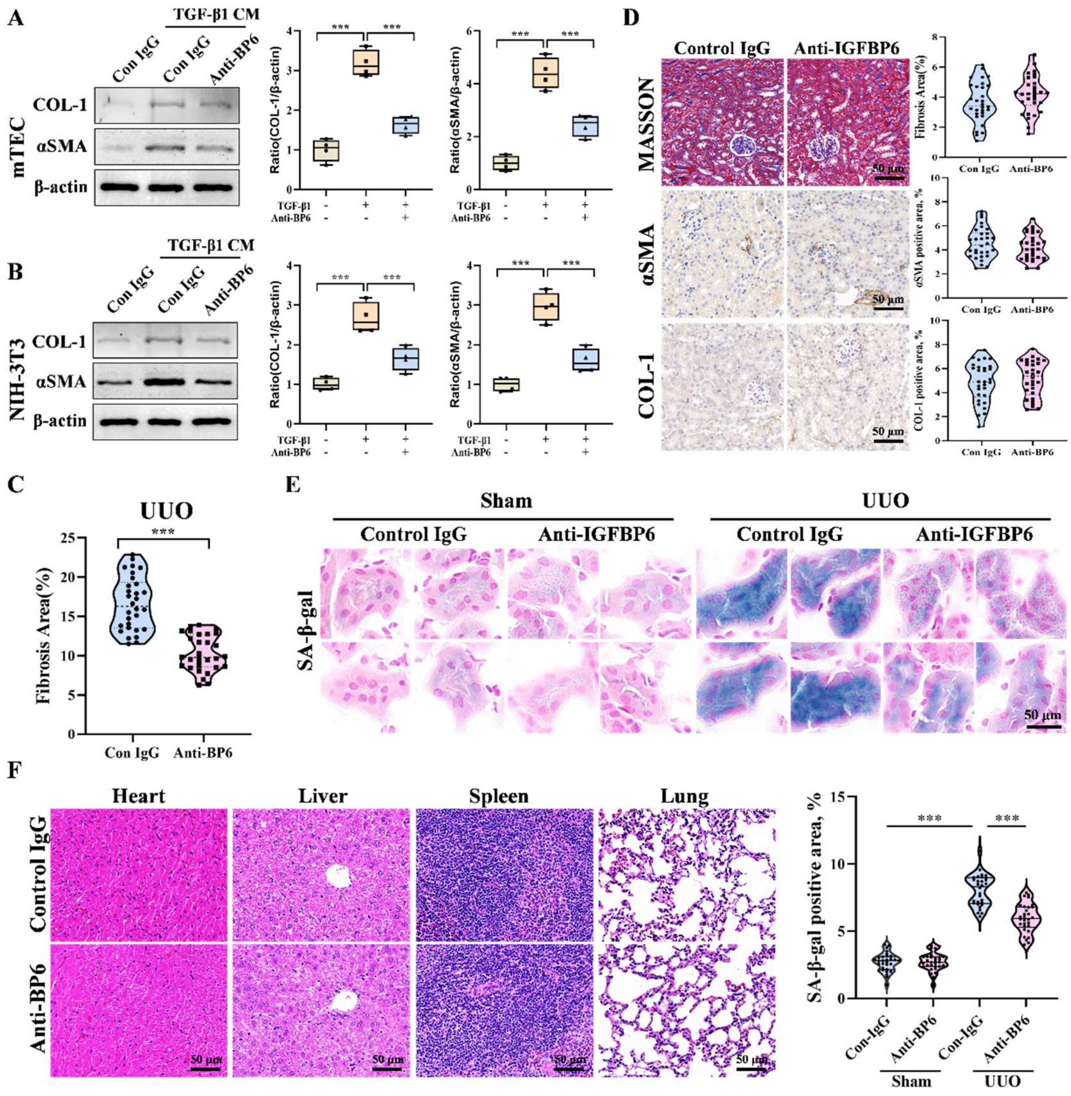

Figure S10. Anti-IGFBP6 treatment can reduce renal cellular senescence and fibrosis.

(A) Western blot and quantification analyses of αSMA and COL-1 in mTEC cells. (B) Western blot and quantification analyses of αSMA and COL-1 in NIH-3T3 cells. (C) The quantification analyses of Masson staining in UUO mice model with or without anti-IGFBP6 treatment (n = 30 views of 6 mice/group). (D) The Masson staining and immunohistochemistry of αSMA and COL-1 in mice with or without anti-IGFBP6 treatment (n = 30 views of 6 mice/group). (E) The SA-β-gal staining in UUO mice model with or without anti-IGFBP6 treatment (n = 30 views of 6 mice/group). (F) HE

staining of the heart, liver, spleen, and lungs after treatment with Control IgG or Anti-IGFBP6. Data are presented as mean ± SEM from at least 3 independent *in vitro* experiments and 6 mice *in vivo*. Statistical differences were determined using an independent sample t-test and one-way ANOVA with Tukey's post hoc analysis. ***P < 0.001.

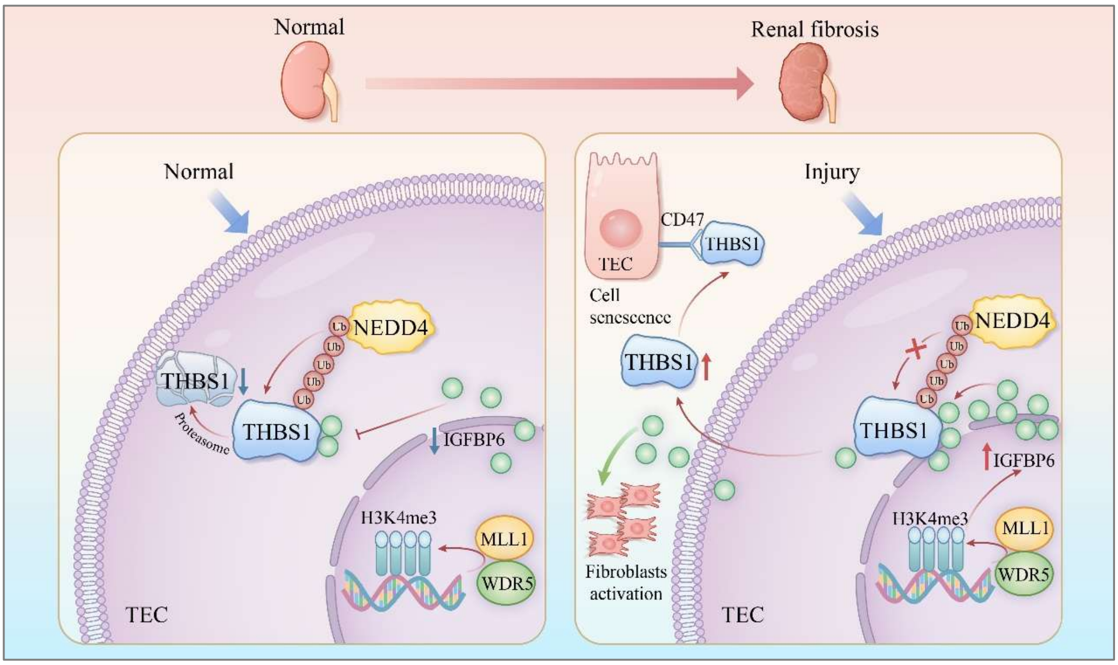

Figure S11. IGFBP6 promotes renal fibrosis by enhancing THBS1/CD47-mediated cell senescence.

Table S1: Clinical features of patients with chronic kidney disease in this study

Gender	Age, years	eGFR (mL/min/1.73m3)	Stage of chronic kidney disease
male	35	111	Stage 1
male	55	109	Stage 1
female	59	102	Stage 1
male	56	111	Stage 1
male	72	95	Stage 1
male	52	119	Stage 1
female	65	69	Stage 2
male	41	70	Stage 2
male	44	80	Stage 2
male	53	76	Stage 2
male	50	75	Stage 2
male	48	78	Stage 2
male	69	81	Stage 2
male	59	88	Stage 2
male	79	87	Stage 2
male	43	64	Stage 2
male	56	64	Stage 2
male	39	88	Stage 2
male	53	69	Stage 2

female	73	79	Stage 2
male	63	67	Stage 2
male	56	66	Stage 2
male	52	82	Stage 2
male	37	87	Stage 2
male	50	41	Stage 3
female	59	41	Stage 3
male	73	47	Stage 3
male	32	40	Stage 3
male	51	32	Stage 3
male	32	58	Stage 3
male	31	57	Stage 3
male	84	20	Stage 4
male	67	29	Stage 4
female	63	33	Stage 4
female	77	34	Stage 4
female	64	33	Stage 4
male	81	19	Stage 4
male	52	16	Stage 4
female	74	29	Stage 4
male	87	8	Stage 5
female	39	13	Stage 5

female	50	5	Stage 5
female	28	8	Stage 5
male	34	9	Stage 5
female	41	9	Stage 5
male	39	5	Stage 5
male	46	4	Stage 5
female	32	7	Stage 5
male	61	4	Stage 5
male	54	6	Stage 5
female	71	11	Stage 5
male	56	9	Stage 5
male	48	6	Stage 5
male	41	5	Stage 5
male	50	6	Stage 5
male	50	6	Stage 5
male	84	8	Stage 5
male	72	13	Stage 5
female	51	10	Stage 5
male	58	14	Stage 5
male	57	7	Stage 5

Note: eGFR: estimated glomerular filtration rate; Stage1: eGFR>90, Stage2: 60<eGFR<89, Stage3: 30<eGFR<59, Stage4: 15<eGFR<29, Stage5: eGFR<15 or renal dialysis.

Table S2: Primers and RNA sequences used in this study.

2. 1 Primers for qRT−PCR (mouse)

Terms	Forward primer (5'-3')	Reverse primer (5'-3')
IGFBP6	CCGCAGACACTTGGATTCAG	GACACTGCTGCTTTCGGTAG
MLL1	AAGGGAACTTCTGCCCTCTC	CGTGTAGGCCACACTTTCTG
WDR5	TATGATGGCCTCTGCCGAAT	TGCAGCCAGGATGTATTTGC
TGF-β1	CCACCTGCAAGACCATCGAC	CTGGCGAGCCTTAGTTTGGAC
IL1β	GAAATGCCACCTTTTGACAGTG	TGGATGCTCTCATCAGGACAG
IL6	CTGCAAGAGACTTCCATCCAG	AGTGGTATAGACAGGTCTGTTGG
CXCL1	ACTGCACCCAAACCGAAGTC	TGGGGACACCTTTTAGCATCTT
CXCL2	CCAACCACCAGGCTACAGG	GCGTCACACTCAAGCTCTG
CXCL10	CCAAGTGCTGCCGTCATTTTC	GGCTCGCAGGGATGATTTCAA
CCL2	TTAAAAACCTGGATCGGAACCAA	GCATTAGCTTCAGATTTACGGGT
CCL20	GCCTCTCGTACATACAGACGC	CCAGTTCTGCTTTGGATCAGC
CSF1	GTGTCAGAACACTGTAGCCAC	TCAAAGGCAATCTGGCATGAAG
MMP7	CTTACCTCGGATCGTAGTGGA	CCCCAACTAACCCTCTTGAAGT
MMP9	GCAGAGGCATACTTGTACCG	TGATGTTATGATGGTCCCACTTG
COL-1	GCTCCTCTTAGGGGCCACT	CCACGTCTCACCATTGGGG
αSMA	CGGGCTTTGCTGGTGATG	CCCTCGATGGATGGGAAA
β-actin	CATTGCTGACAGGATGCAGAA	ATGGTGCTAGGAGCCAGAGC

2. 2 Primers for qRT−PCR (rat)

Terms	Forward primer (5'-3')	Reverse primer (5'-3')
COL-1	TGACTGGAAGAGCGGAGAGT	GATAGCGACATCGGCAGGAT
αSMA	GGAGCATCCGACCTTGCTAA	AGAGTCCAGCACAATACCAGTT
β-actin	ATATCGCTGCGCTCGTCGT	CATACCCACCATCACACCCTGG

2. 3 Primers for qRT−PCR (human)

Terms	Forward primer (5'-3')	Reverse primer (5'-3')
IGFBP6	TGCCGTAGACATCTGGACTC	GGGCACGTAGAGTGTTTGAG
MLL1	AAGAGCAGGTAAACTCTCTCCTC	TTCCTCTCCGTCGTACAATTTG
WDR5	AATTCAGCCCGAATGGAGAGT	AGGCTACATCGGATATTCCCAG
β-actin	CATGTACGTTGCTATCCAGGC	CTCCTTAATGTCACGCACGAT
